# Multiparametric Classification Links Tumor Microenvironments with Tumor Cell Phenotype

**DOI:** 10.1371/journal.pbio.1001995

**Published:** 2014-11-11

**Authors:** Bojana Gligorijevic, Aviv Bergman, John Condeelis

**Affiliations:** 1 Department of Systems and Computational Biology, Albert Einstein College of Medicine, Bronx, New York, United States of America; 2 Gruss-Lipper Biophotonic Center, Albert Einstein College of Medicine, Bronx, New York, United States of America; 3 Department of Anatomy and Structural Biology, Albert Einstein College of Medicine, Bronx, New York, United States of America; Glasgow, United Kingdom

## Abstract

While it has been established that a number of microenvironment components can affect the likelihood of metastasis, the link between microenvironment and tumor cell phenotypes is poorly understood. Here we have examined microenvironment control over two different tumor cell motility phenotypes required for metastasis. By high-resolution multiphoton microscopy of mammary carcinoma in mice, we detected two phenotypes of motile tumor cells, different in locomotion speed. Only slower tumor cells exhibited protrusions with molecular, morphological, and functional characteristics associated with invadopodia. Each region in the primary tumor exhibited either fast- or slow-locomotion. To understand how the tumor microenvironment controls invadopodium formation and tumor cell locomotion, we systematically analyzed components of the microenvironment previously associated with cell invasion and migration. No single microenvironmental property was able to predict the locations of tumor cell phenotypes in the tumor if used in isolation or combined linearly. To solve this, we utilized the support vector machine (SVM) algorithm to classify phenotypes in a nonlinear fashion. This approach identified conditions that promoted either motility phenotype. We then demonstrated that varying one of the conditions may change tumor cell behavior only in a context-dependent manner. In addition, to establish the link between phenotypes and cell fates, we photoconverted and monitored the fate of tumor cells in different microenvironments, finding that only tumor cells in the invadopodium-rich microenvironments degraded extracellular matrix (ECM) and disseminated. The number of invadopodia positively correlated with degradation, while the inhibiting metalloproteases eliminated degradation and lung metastasis, consistent with a direct link among invadopodia, ECM degradation, and metastasis. We have detected and characterized two phenotypes of motile tumor cells *in vivo*, which occurred in spatially distinct microenvironments of primary tumors. We show how machine-learning analysis can classify heterogeneous microenvironments *in vivo* to enable prediction of motility phenotypes and tumor cell fate. The ability to predict the locations of tumor cell behavior leading to metastasis in breast cancer models may lead towards understanding the heterogeneity of response to treatment.

## Abstract 2

Tumor microenvironment features are established as predictors of tumor cell behavior and fate.

## Author Summary

A large proportion of cancer deaths are due to metastasis—the spread of cancer from the primary tumor to other parts of the body. Movement of cells may require the formation of protrusions called invadopodia, which degrade extracellular matrix. Although some studies have reported on locomotion in primary tumors, the presence of invadopodia was not tested. Here, we show that single cells from mouse mammary carcinoma can move using a fast- or slow-locomotion mode depending on different levels of cues present in the tumor microenvironment. Using multiphoton microscopy *in vivo* combined with a machine-learning algorithm we show how manipulation of microenvironmental conditions can induce predictable changes in the number of locomoting cells or switch between the two locomotion modes. We also demonstrate that only the slower moving cells are associated with invadopodia *in vivo*, and that only tumor cells from regions rich in invadopodia degrade the surrounding extracellular matrix and disseminate. Specific targeting of invadopodia results in inhibition of lung metastasis. This work proposes a systems biology view of how tumor microenvironments regulate tumor progression and presents insight into the heterogeneity of the treatment response. The ability to define and predict conditions under which tumor cells disseminate offers potential therapeutic benefits in regulating tumor progression.

## Introduction

Statistics from 2013 show that more than 90% of breast cancer deaths are a consequence of metastatic disease and that median survival for metastatic breast cancer is 3 years [1]. Accordingly, a better understanding of the physiological conditions that help initiate or facilitate metastatic recurrence would be valuable for identifying and improving outcomes for patients with metastatic breast cancer. The process of metastasis [2] includes epithelial-mesenchymal transition, tumor cell locomotion through the interstitial matrix, dissemination from the primary tumor via vasculature (intravasation and extravasation), and seeding of secondary organs [3]. The chemical signals that initiate and direct tumor cell motility are typically growth factors secreted either by host cells in the primary tumor microenvironment (e.g., macrophages, endothelial cells, fibroblasts, and pericytes) or by tumor cells themselves [4], and may be present either in soluble form or bound to the extracellular matrix (ECM). Recently, increasing evidence has shown that mechanical signals in tumor microenvironments may also modify tumor cell behavior. Tumor cell locomotion has been shown to correlate with proximity to macrophages [5] and blood vessels [6] while increased cross-linking [7], stiffness [8],[9], and alignment [10] of the ECM correlate with increased metastasis.

In summary, many studies have investigated individual tumor cell–stromal cell or tumor cell–ECM interactions, revealing a number of possible sources of chemo- and hapto-tactic signals for tumor cells, as well as the high level of complexity in the tumor microenvironment network [4]. Moreover, a number of tumor microenvironment studies [11] have shown positive or negative effects of stromal cell types or individual mechanical parameters on tumor cell motility within the primary tumor and subsequent metastasis. However, an integrated view of the tumor microenvironment as a complex system, including how all relevant biological players in combination affect tumor cell motility and metastatic outcome, is still missing.

In ECM that is soft, porous, and with low levels of cross-linking, single tumor cells can locomote quickly and in a metalloprotease (MMP)-independent manner [12] by utilizing forces generated by adhesion and actin polymerization [13],[14]. Locomotory protrusions (pseudopodia) form at the front of the cell, followed by locomotion and translocation of the entire cell. Stiff, non-compliant ECM contains high concentrations of cross-linked collagen and/or basement membrane proteins. To enable movement, tumor cells remodel their immediate ECM, mainly via the degradative action of MMPs [12],[15],[16]. Studies in cell culture models have shown that, when grown on ECM in the presence of growth factors, metastatic cancer cells form invadopodia [17]. These are dynamic membrane protrusions that are enriched in actin, actin regulatory proteins, cortactin, tyrosine kinases, Tks5, and proteases [18], and are ECM-degrading structures. *In vitro*, invadopodia form in 10%–20% of tumor cells plated on ECM [19], and their average size in the light microscope is approximately 1–3 µm in diameter and 2–12 µm in length, depending on ECM density and dimensionality (2-D versus 3-D) [19]–[21].

The invadopodium compartment is simultaneously the site of actin polymerization, cell contact to ECM, and matrix proteolysis; thus, the invadopodial lifetime is on the scale of hours, significantly longer than any other type of membrane protrusions in tumor cells [19],[22]. Although a large part of the invadopodial signaling network overlaps with that of other membrane structures such as lamellipodia, filopodia, and focal adhesions, the defining feature of invadopodia is their high proteolytic activity, which degrades and remodels ECM. This suggests that invadopodia may be a key mechanism for MMP-dependent tumor cell invasion [23].

Recent studies have gone beyond 2-D and into the 3-D realm of studying invadopodia [20] and podosomes [24]. Moreover, there are recent attempts to study these structures *in situ*, in their natural environment, including podosomes in mouse aorta [25], invadopodia in dermis [26], as well as protrusions similar to invadopodia in organogenesis models of *Caenorhabditis elegans* [27] and *C. intestinalis* [28]. Further, we have recently shown a direct link between the ability of tumor cells to assemble invadopodia and degrade matrix using MMPs *in vitro* and *in situ* and the ability to intravasate and metastasize *in vivo* [29],[30]. Collective data from these and similar studies demonstrate that morphology and protein distributions of invadopodia and podosomes depend on the matrix dimensionality, architecture, and complexity as well as the cell line/type. Neither tumor cells nor the structures they assemble appear the same in 2-D or 3-D [20] culture, or within the fixed tissue [29]. Hence, to identify podosomes and/or invadopodia in different microenvironments and under new experimental conditions, a combination of defining features such as small size, structural components (e.g., Tks5, cortactin) and functionality (MMP-dependent ECM-degradation) may be used, classifying structures as podosomes when referring to structures in myelomonocytic, endothelial, and vascular smooth muscle cells [17] and invadopodia when referring to tumor cells.

Although tumor cell locomotion in primary tumors has been observed in a number of studies [4],[5],[31]–[34], the exact location and timing of invadopodium formation is not presently known. Combined results from numerous studies using preparations of collagen I (with or without cross-linking), collagen IV, laminin, fibrin, chorioallantoic, and peritoneal membranes [12],[35] converge on the conclusion that invadopodia have the ability to degrade and remodel different ECM components present in interstitial tissue and basement membranes of metastatic tumor models [29]. However, where and when invadopodia form in the primary tumor *in vivo* has yet to be determined. Here, we have characterized and quantified two motility phenotypes occurring in primary breast tumors *in vivo*: fast-locomotion and slow-locomotion associated with invadopodia. We show that invadopodia (and slow-locomotion) occur in regions of primary tumor that are spatially distinct from regions where fast-locomotion is the dominant phenotype. We further combined intravital multiphoton microscopy, image analysis, and multiparametric data classification, to analyze microenvironmental conditions under which tumor cells move via either phenotype. We found that the prediction of primary tumor locations where tumor cells locomote fast or form invadopodia and locomote slowly cannot be achieved by any individual tumor microenvironment parameters but only by their combination.

## Results

**Figure 1:**
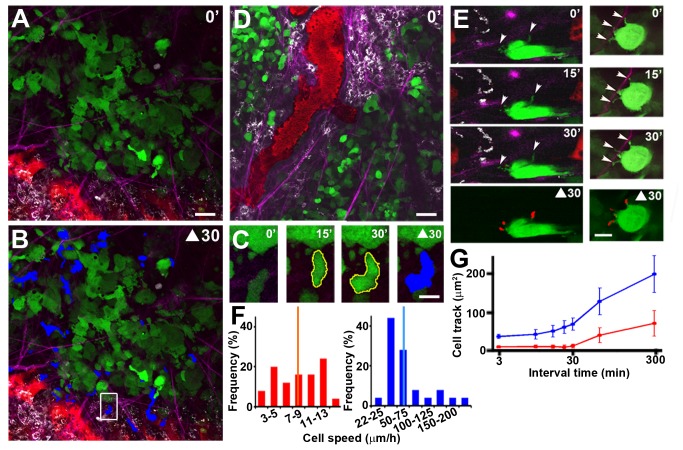
Mammary breast carcinoma exhibits two motility phenotypes. (A) Locomotion of MDA-MB-231-Dendra2 tumor cells (green) is analyzed in each section of 4-D stack from 0′–30′, while simultaneously visualizing collagen fibers (purple), blood vessels (red), and macrophages (white). Scale bar: 60 µm. See Movie S1a. (B) Cell tracks over the 30′ period (30′–0′ = Δ30) are overlayed in blue showing fast locomotion in a number of tumor cells. (C) Zoom-in into an individual tumor cell (yellow outline) at 0′, 15′, and 30′ and the cell track over 30′ period (blue overlay). Scale bars: 10 µm. (D) Example of a field of view where fast locomotion was not detected. Scale bar: 60 µm. See Movie 1b. (E) Zoom-in into two different tumor cells shows that they have small protrusions (white arrowheads). Still frames at 0′, 15′, and 30′ are shown. Bottom panels show motility over 30′ (Δ30, red), overlaid on image at 0′. Also see Movie S2. Scale bars: 10 µm. (F) The two phenotypes of motility can be distinguished by comparing cell velocities of cell front, measured as average distance per hour. Tumor cells exhibit either small protrusions associated with slow-locomotion (red bars, velocity range 2–15 µm/h, red line is the mean, 8 µm/h) or fast-locomotion (blue bars, velocity range 22–250 µm/h, blue line is the mean, 69 µm/h). Note the difference in the bin range. For legibility, interval of every second bar is listed. (G) Comparisons of cell tracks over 300 min. Measurements were done at 3′, 9′, 15′, 21′, 30′, 60′, and 300′; time is plotted on a log scale. Each time point represents mean ± SEM. Measurements were done using 78 tumor cells in *n* = 7 animals.

By recording 4-D stacks in orthotopic xenograft tumors formed from a metastatic, breast cancer cell line MDA-MB-231-Dendra2, we confirmed the presence of a tumor cell behavior previously described in detail, where cells quickly locomote through the tissue either individually or in streams (Figure 1A–1C; Movie S1a) [4],[31]–[34]. Subtraction of the initial frame (0′) from that taken at 30 min (30′) in a single z-section, detects the parts of the cell that moved over 30 min and the area the cell covered (Δ30 cell tracks) (Figure 1B, blue). However, in some fields of view, tumor cells exhibited a different phenotype. Fast-locomotion was not detected (Figure 1D and 1E; Movie S1b) but was replaced by slow-locomotion. Imaging at high magnification revealed the presence of small protrusions (Figure 1E, white arrowheads and red cell tracks; Movie S2), often adjacent to collagen fibers (Movie S2a and S2b), macrophages and blood vessels (Movie S2c and S2d). Longer time-lapse imaging showed that tumor cells with small protrusions exhibited a slower locomotion phenotype Figure 1F and 1G, red) in comparison to previously reported fast-locomotion (Figure 1F and 1G, blue). Cells migrating with velocities of 22–250 µm/h were classified as fast-locomoting, while those with small protrusions and velocities 2–15 µm/h were classified as slow-locomoting (Figure 1F).

**Figure 2:**
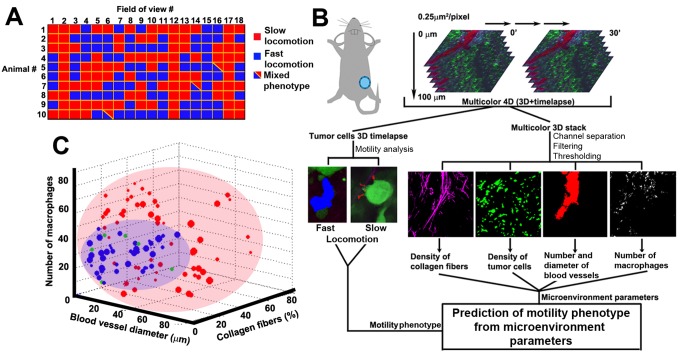
Tumor microenvironment complexity requires a non-linear SVM classification to predict locations of motility phenotypes. (A) Fast-locomotion (blue squares) or small protrusions associated with slow-locomotion (red squares) were scored in 184 fields of view collected in ten animals. These two phenotypes were mutually exclusive in 181/184 fields of view (blue and red squares). (B) Workflow of the intravital systems microscopy approach: The mammary imaging window is surgically implanted on top of the tumor and multicolor/4-D stacks (512 µm×512 µm×100 µm×30′) are collected in one to six random locations, with maximum 3 h of imaging. 3-D time lapses of motile tumor cells (green channel) are separated into fast-or slow-locomotion on the basis of cell track size. Multicolor 3-D stacks (at a single time point) are separated into four channels and microenvironment parameters were extracted. Next, statistical analyses are done to link motility phenotypes with microenvironment parameters. (C) A 3-D projection with top three performing parameters from 5-parameter SVM classification of tumor microenvironments. Circle sizes reflect the number of motile cells detected in a field of view. Shaded areas illustrate 3-D “phase space” of two microenvironment classes. Misclassifications (green circles) occur at borderline conditions. Also see Figure S1 and Movie S3.

Systematic analysis of fields of view in ten animals has shown that the two motility phenotypes were usually mutually exclusive, i.e., tumor cells within a specific field of view exhibited only one of the observed phenotypes at a time in 184/187 fields (Figure 2A). As xenograft tumors consist of genetically similar tumor cells, we hypothesized that the presence of two motility phenotypes is controlled via the tumor microenvironment and may be distinguished by different levels of one or more microenvironmental factors, whose measurement can be then used as a predictor of tumor cell behavior.

To investigate which microenvironmental conditions are amenable for either motility phenotype, we monitored tumor cell phenotype relative to the number of microenvironment parameters previously reported to influence tumor cell locomotion (Figure 2B) [5]–[10]. These included the density of collagen fibers [7]–[10], tumor cells, and macrophages [5] as well as the number and size of blood vessels [6] present in the field of view. We tested each of the parameters individually for their correlation with either of the motility phenotypes, attempting to find values of a single parameter or combination of them that coincide with one of the two phenotypes. However, none of the commonly used statistical methods based on linear separation (model selection, partial correlation, or causal modeling) (Figure S1A–S1C) were able to provide a model capable of distinguishing between microenvironment parameters and the presence of different motility phenotypes. A possible reason for the lack of linear predictors is the existence of complex interactions among parameters, such as those present in the tumor microenvironment [4],[11]. To test this, we employed support vector machine (SVM), an algorithm suitable for segmentation of non-linearly separable data, such as the data in Figure S1A. Intuitively, the SVM algorithm “converts” a non-linearly separable dataset into linearly separable set by increasing the dimensionality of the hyperspace (initially, N parameters create hyperspace of N dimensions). For illustrative purposes, see a one-dimensional example in Figure S11A.

In order to link between microenvironmental parameters and tumor cell locomotion phenotypes, we used microenvironmental parameters as an “input” for the SVM classifier, with fast-locomotion (blue dots) or slow-locomotion (red dots) as the classifier's “output” (Figure S1D). Classification was done on the basis of three, four, or five parameters with increasing accuracy, reaching maximum accuracy of 92% when all five parameters were used (Figure S1D).

Figure 2C shows a 3-D projection of the hyperspace with maximum accuracy classification. The high accuracy of the SVM classification (and cross-validation) means that it is possible to predict whether tumor cells will locomote fast or slow (output) on the basis of a given set of microenvironmental parameters (input). By utilizing only the microenvironmental parameters within a field of view as input information, we get a prediction of the motility phenotype present in that same field of view, which is output information. Misclassification (green dots) probability of <8% (and <5% when training includes the entire dataset) is associated mainly with parameter values on the classification border between red space and blue space (Figure 2C, red- and blue-shaded areas). None of these individual microenvironment parameters alone offers a sufficient predictor, as their contribution to motility phenotype depends on the context created by all other microenvironment parameters. Only a nonlinear transformation of all parameters could distinguish between microenvironments associated with either motility phenotype (Figure S1). In conclusion, tumor motility phenotypes can be distinguished only by a non-linear, multiparametric analysis such as SVM, as they are a result of balance among multiple signals within complex tumor microenvironments.

**Figure 3:**
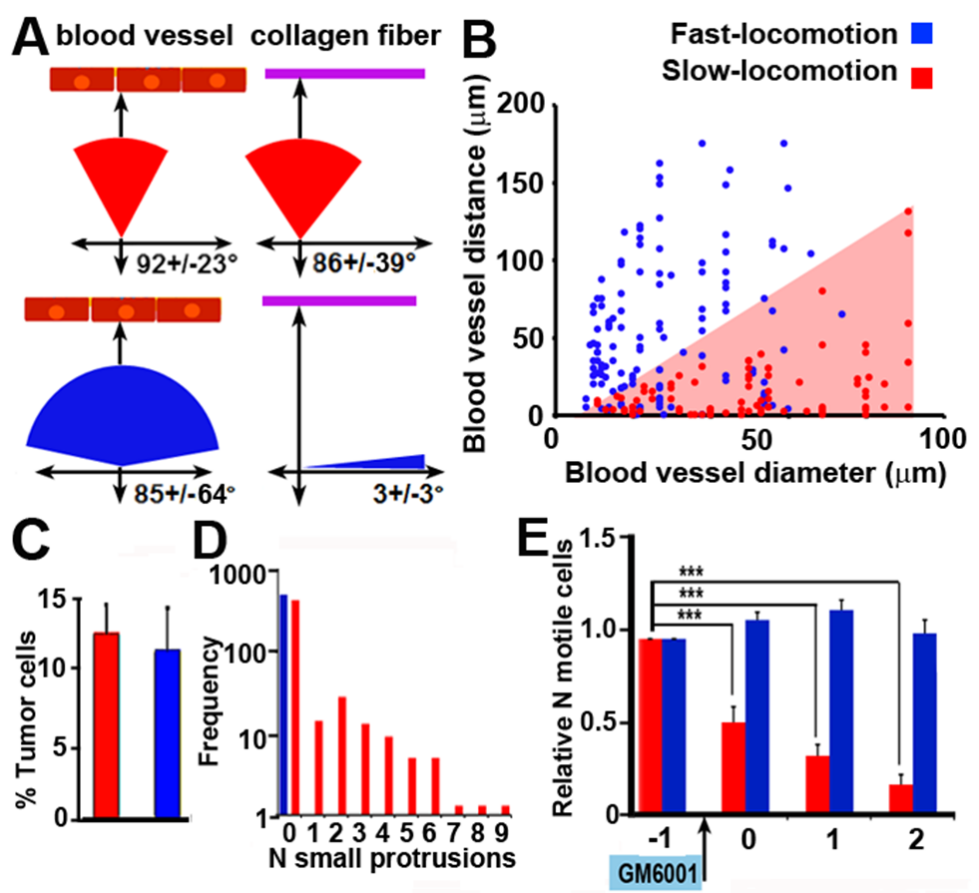
Small protrusions associated with slow-locomoting cells are directed at ECM and blood vessels. (A) Range of directions of cell motility in relation to closest blood vessel (red strip) or collagen fiber (purple line). Protrusions on slow-locomoting cells are commonly directed towards blood vessels and surrounding collagen fibers while the direction of fast-locomoting cells is along collagen fibers and independent of blood vessels. (B) Fast-locomotion (blue dots) is independent on the blood vessel size. In contrast, the ability of distant tumor cells to form small protrusions is dependent on the blood vessel diameter. Tumor cells can form small protrusions either close or far from macrovessels, but only adjacent to microvessels (red shaded triangle illustrates this trend). While cells with small protrusions show a significant positive trend in variance (R^2^ = 0.29, *p* = 1.7×10^−8^), distance of fast-locomoting cells' from the blood vessel is independent on the blood vessel diameter (R^2^ = 0.07, *p* = 1.5×10^−3^). See Materials and Methods for details. (C) Small protrusions associated with slow-locomotion (red) and fast-locomoting cells (blue) occur in similar percent of tumor cells within respective field of view (measured over 30′). (D) Numbers of small protrusions present on each of the fast- or slow- locomoting cells. Fast-locomoting cells do not exhibit small protrusions. Measurements were based on 1,000 cells in *n* = 4 animals and plotted on log scale. (E) Injection with MMP-inhibitor GM6001 (arrow) does not affect fast-locomotion (blue) but eliminates small protrusions (red). Measurements are based on 15 time-lapse movies in *n* = 3 animals, (***p<0.001, based on paired *t*-tests, bars represent means ± SEM).

To better understand the link between the tumor microenvironment and tumor cell motility phenotypes, we analyzed each of the motility phenotypes at single cell level (Figure 3). We characterized the relationship between individual tumor cells of either phenotype and landmarks within the tumor microenvironment (Figure 3A). While directionality of fast-locomotion seemed to be controlled by the orientation of adjacent collagen fibers (Figure 3A, blue pie charts), the small protrusions in slow-locomoting cells were commonly perpendicular to adjacent blood vessels and collagen fibers that surround blood vessels (Figure 3A, red pie charts). To further analyze the relationship between blood vessels and small protrusions, we compared the distance of tumor cells with either motility phenotype to the nearest blood vessel and the size of that blood vessel (Figure 3B). Our results show that fast-locomotion may occur far from blood vessels (21/132 of fast-locomoting cells were found>100 µm from the nearest blood vessel) and is independent of the blood vessel size. However, the distances from the nearest vessel at which small protrusions formed were smaller (2/95 of slow-locomoting cells were found>100 µm from the nearest blood vessel) and positively correlated with vessel diameter (Figure 3B, red triangle), suggesting that small protrusions are most likely initiated by a chemical or mechanical gradient associated with the blood vessel. We also measured the amount of ECM components not detected by second harmonic generation (Figure S2), demonstrating that deposits of basement membrane proteins laminin (Figure S2A) and collagen IV (Figure S2B) increase with blood vessel size, suggesting that small protrusions are associated with larger blood vessels with this matrix composition.

We next looked at the number of motile cells within fields of view where either fast- or slow-locomotion was detected. Both fast (blue bars) and slow (red bars) locomotion each occurred in approximately 15% of tumor cells in the field of view (Figure 3C) and only slow-locomoting cells exhibited small protrusions (Figure 3D). Finally, small protrusions associated with slow-locomotion were found to be inhibited by tail-vein injection of MMP inhibitor GM6001, suggesting MMP-dependence, while the fast-locomotion remained unaffected throughout the 3 h time lapse (Figure 3E).

**Figure 4:**
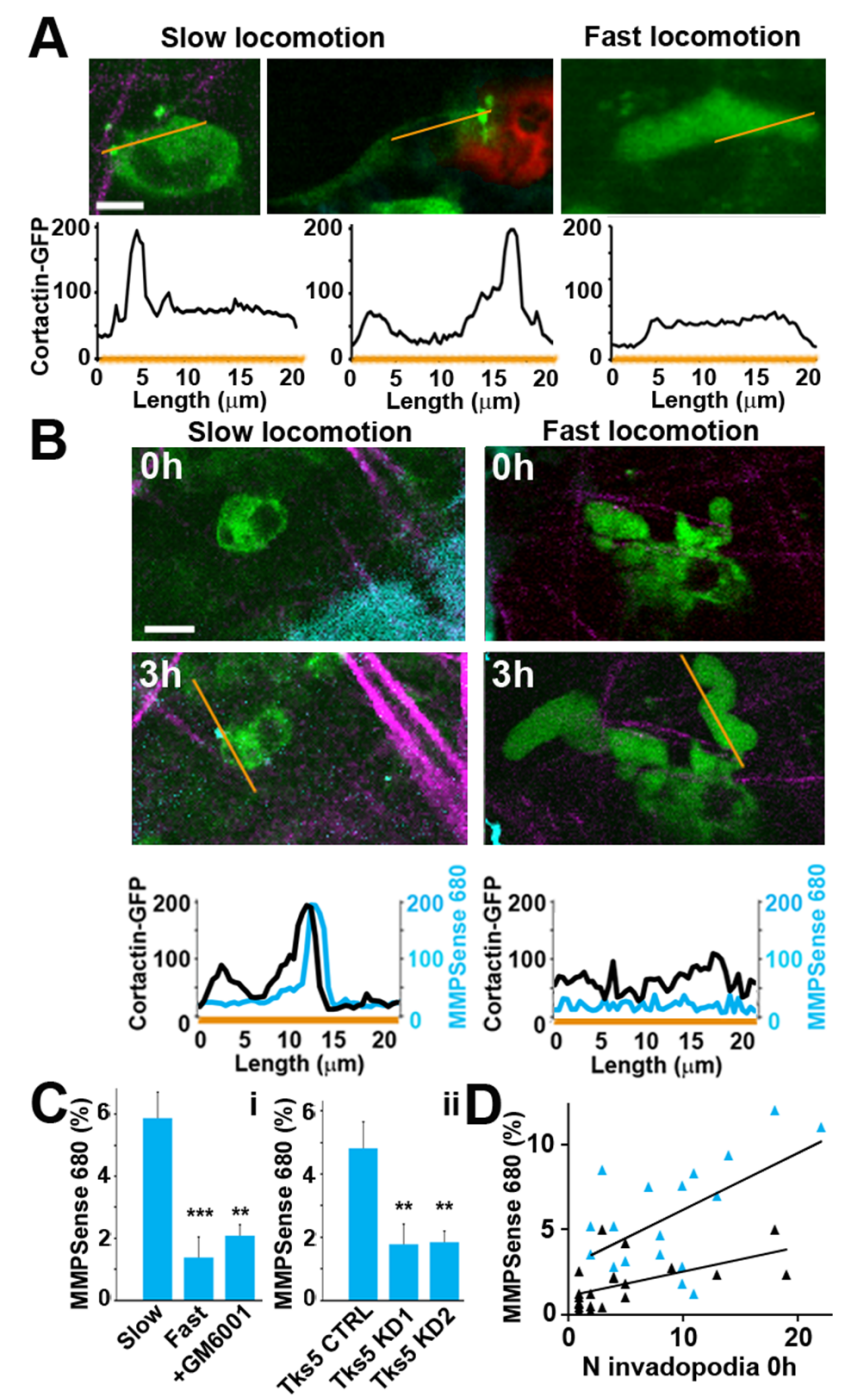
Small protrusions on slow-locomoting cells have the defining characteristics of invadopodia. (A) *In vivo* images of MDA-MB-231-cortactin-GFP cells show enrichment of cortactin (green) at the tip of small protrusions (left and middle panels) adjacent to collagen fibers (purple) or blood vessels (red) and, based on SVM parameter classification, located in microenvironments amenable for slow-locomotion. In the fast-locomoting cells (right panel), cortactin-GFP fluorescence is uniformly distributed (see Movie S4). Lower panels are line-scans of cortactin-GFP fluorescence along the yellow lines. Scale bars: 10 µm. (B) MMPSense 680 can be observed in the blood vessel lumen immediately after injection. After extravasation (approximately 3 h), MMPSense 680 (cyan) colocalizes with the small protrusion (left), but is not present around the fast-locomoting cell (right). Lower panels are line-scans of cortactin-GFP (black) and MMPSense 680 (cyan) along the yellow lines, and show colocalization of cortactin and MMPSense 680 in the small protrusion. Also see Figure S5 and Movie S5. Scale bars: 10 µm. (C) Comparison of MMPSense 680 accumulation (detected at 24 h) in fields of view where either slow-locomotion (“Slow” bar) or fast-locomotion (“Fast”) were detected at 0 h, orin fields of view where slow-locomotion was detected at 0 h prior to treatment with GM6001 (panel i). Accumulation of MMPSense 680 was also measured in Tks5 CTRL tumors and knockdown tumors Tks5 KD1 and Tks5 KD2 (panel ii). Same fields of view were located at 0 h and 24 h using fate-mapping. See Materials and Methods and Figure S6. We observed approximately 3-fold higher relative MMPSense 680 signal in fields of view with slow-locomotion compared to fields of view with fast locomotion or treated with GM6001. Similar reduction in MMPSense 680 signal is observed in Tks5 knockdown tumors. Each bar represents mean ± SEM based on ≥6 regions in *n*≥3 animals. **p<0.01, ***p<0.001, using homoscedastic t-tests. (**D**) We observed significant positive correlation (R^2^ = 0.44, *p* = 6×10^−5^) between the number of small protrusions at 0 h and MMPSense 680 fluorescence measured at 24 h (cyan triangles), but not when animals were treated with GM6001 (black triangles; R^2^ = 0.14, *p* = 0.06).

Our phenomenological data, including morphology, relationship to ECM and blood vessels, as well as MMP-dependence of small protrusions led us to the hypothesis that the observed small protrusions are invadopodia *in vivo*. To test this hypothesis, we used previously established structural and functional markers of invadopodia. Molecularly, invadopodia have been shown to be enriched in active proteases and structural components including cortactin and Tks5 [35]–[39]. While cortactin is present both in invadopodia and at the leading edge of migrating cells in 2-D culture [40], in 3-D conditions it is enriched primarily at the tip of invadopodial protrusions at the cell front [20],[29]. By using MDA-MB-231-cortactin-GFP cells [20], we were able to directly compare cortactin-enriched compartments in 3-D collagen I (Figure S3A), in cryosections (Figure S3B) [29] and *in vivo* (Figure 4). Similarly to 3-D and cryosections, the small protrusions observed *in vivo* (left and middle panels) showed a peak of cortactin fluorescence at the protrusion tip (Figure 4A, yellow lines in upper panels and associated line-scans in lower panels; Movie S4b). In contrast, fast-locomoting cells showed a homogeneous distribution of cortactin throughout the cell (Figure 4A, right panels; Movie S4a). These results are consistent with the identification of the small protrusions as invadopodia *in vivo*.

We next constructed cell lines where the Tks5 structural protein of invadopodia was knockdown (KD) with shRNA, which specifically targets invadopodia in MDA-MB-231 cells and tumors (Figure S4). Tks5 knockdown was previously shown to inhibit invadopodium maturation and degradation [39],[41] in 2-D culture conditions [39] as well as to greatly reduce lung metastases in mouse models [41]. Our data confirmed that Tks5 KD1 and Tks5 KD2 cells (Figure S4A) did not assemble invadopodia *in vitro*, under culture conditions (Figure S4B and S4C), as shown using Tks5/cortactin antibodies and fluorescent gelatin degradation. Similar results were confirmed in primary tumors, where knockdown efficiency was maintained (Figure S4D), and Tks5-positive punctae which colocalized with collagen I degradation (Figure S4E) were present only in TKs5 CTRL tumors but not in knockdowns (Figure S4F). In addition, Tks5 KD1 and Tks5 KD2 tumors *in vivo* did not exhibit small protrusions, while the fast locomotion behavior was only slightly affected (Figure S4G), supporting our hypothesis that small protrusions are indeed invadopodia and they were selectively targeted by Tks5 knockdown.

Finally, to directly test if the small protrusions function as invadopodia *in vivo*, we measured the ECM degradation activity associated with the small protrusions. The antibody against degraded collagen we used for immunohistofluorescence (Figure S3) is unsuitable for *in vivo* use due to the inefficient delivery and labeling. Instead, we used the MMP-activated substrate MMPSense 680 (Perkin Elmer) for intravital imaging [42]. To validate this reporter, we compared ECM degradation as measured by MMPSense 680 solution (cyan) and a more commonly used substrate, DQ-collagen I gel (red) [14],[43] in 3-D culture of cortactin-TagRFP cells (green) (Figure S5A and S5B). Quantitation of ECM degradation area with or without MMP inhibitor GM6001 (Figure S5C) showed similar trends with both reporters. *In vivo*, MMPSense 680 (cyan) injected into MDA-MB-231-cortactin-GFP tumors (green) colocalized with cortactin-enriched protrusions after 3 h (Figure 4B, left panels; yellow lines correspond to line-scans; Movie S5a). In contrast, fast-locomoting cells did not colocalize with MMPSense 680 (Figure 4B, right panels; Movie S5b). Finally, we monitored the accumulation of MMPSense 680 signal in microenvironments where small protrusions were either present or absent, in microenvironments treated with GM6001, as well as in Tks5 knockdown tumors. This experiment was done using photoconversion followed by MMPSense 680 injection and image collection from 0–96 h (Figure S6). We quantified MMPSense 680 signal (Figure 4C) at 24 h, when the signal in the tumor plateaus. In fields of view where small protrusions were present, we measured that 6% of image area contained MMPSense 680 fluorescence above threshold, reflecting the locations of MMP-based degradation and this was approximately 3-fold higher than in other areas, GM6001-treated areas or knockdown tumors. In addition, MMPSense 680 fluorescence was positively correlated with the number of small protrusions (Figure 4D), and this correlation was eliminated by GM6001 treatment. As all our results are consistent with the hypothesis that the small protrusions are in fact invadopodia *in vivo*, we will henceforth refer to them as invadopodia.

**Figure 5:**
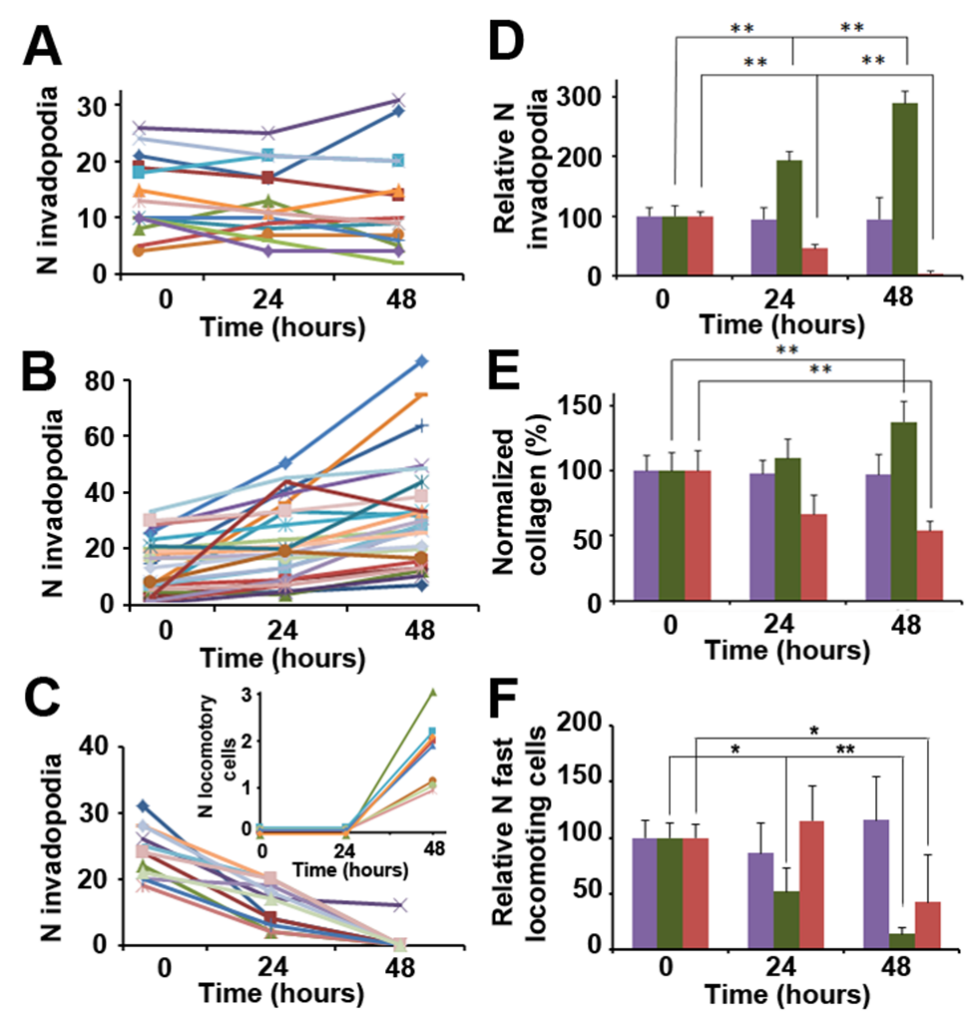
ECM modulation changes the frequency of motility phenotypes and may induce phenotype switching. (A–C) The number of invadopodia monitored in matched fields of view remains stable over 2 days. Each field of view is represented by different line color. (B) Invadopodium number is significantly increased by 1 g/kg/day L-ribose treatment. (C) Invadopodium number is diminished by 6 mg/kg/day BAPN treatment. Inset: some fields of view start exhibiting fast-locomotion phenotype when invadopodia are diminished. Such transition correlates with loosening of the ECM. (D) Quantification of raw data from (A–C). Normalized numbers of invadopodia show no significant change in control areas 0–48 h (purple bars). A significant increase follows L-ribose treatment (green bars, *p*<10^−3^) and a significant decrease follows BAPN treatment (red bars, *p*<10^−4^) based on paired *t*-tests. Average values along with ±SEM are shown. (E) Normalized collagen fiber density, measured by second harmonic in the same areas as (D), follows the same trends as invadopodia numbers. While control areas show minimal collagen remodeling over 48 h, treatment with L-ribose (green bars) increases cross-linking and fiber bundling, resulting in fiber density increase, while BAPN treatment (red bars) reduces cross-linking and results in lower fiber density at 48 h. Also See Figure S8. (F) Numbers of fast locomoting cells show no significant change in control areas 0–48 h (purple bars), while L-ribose treatment (green bars) induces significant reduction of fast locomotion. In addition, BAPN treatment (red bars) induces reduction of fast locomotion at 48 h. Average values along with ±SEM are shown, paired *t*-tests were done for comparison, *p<0.05, **p<0.01.

The results of SVM classification show that the two motility phenotypes, which we now recognized as fast-locomotion and invadopodia-associated slow-locomotion, exist in distinct yet neighboring conditions. Such a result suggests that a shift along one of the axes in the 3-D plot in Figure 2C may induce a change in the number of tumor cells exhibiting either motility phenotype, i.e., induce a phenotype switch. The changes in tumor cell behavior are context-dependent, which means that the extent of the influence of one parameter over the phenotype, or its capability to cause a phenotype switch, depends on the context of other parameters (Figure S7). We hypothesized that a motility phenotype switch in primary tumors may occur, for example, when collagen fiber density changes over time. An increase in collagen fiber density may be initiated because of fibroblast deposition of collagen, while decreases may be a result of the invadopodium-mediated degradation of fibers. Such logic is strengthened by previous *in vitro* reports showing that both the speed of MMP-dependent 3-D migration [44] and the number of invadopodia in 2-D assays are controlled by the rigidity and cross-linking level in basement membrane extracts, collagen, and synthetic matrices [45],[46]. We tested the effect of ECM rigidity/cross-linking by modulating ECM cross-linking levels and measuring the number of invadopodia, which are associated with slow-locomotion phenotype (Figure 5). In the control set of animals, we imaged the same fields of view (using photoconversion to match fields over time) at 0, 24, and 48 h, demonstrating that invadopodia are present over the entire period under control conditions (Figure 5A). Collagen imaging confirmed that over a 48 h period, collagen I fibers remained stable with minor changes (Figures 5E, purple bars, and S8A). A different set of animals was treated with L-ribose, which was shown to increase cross-linking and hence stiffness in collagen-based gels [7]. A considerable increase in the number of invadopodia (Figure 5B) was detected, accompanied by an increase in collagen I density (Figures 5E, green bars, and S8B). The third set of animals was treated with the lysil oxidase (LOX) inhibitor β-aminopropionitrile (BAPN), which reduces cross-linking and loosens the ECM [7]. Treatment with BAPN reduced and finally eliminated invadopodia (Figure 5C). Figure 5D shows that invadopodium number follows the trends in collagen fiber density. Moreover, as a result of fibrillar collagen I density decrease (Figures 5E, red bars, and S8C), in several fields of view, tumor cells switched from invadopodium-associated slow-locomotion at 0 h to fast-locomotion at 48 h in the same field of view (Figure 5C, inset).

Areas with fast-locomotion also showed a dramatic reduction of fast-locomoting cells following the L-ribose treatment (Figure 5F, green bars) accompanied by the appearance of invadopodia. Interestingly, BAPN treatments also induced a slight decrease in the number of fast-locomoting cells over 48 h (Figure 5F, red bars). One of the possible explanations may be the lack of balance between adhesion and traction forces at low ECM stiffness levels, which was previously shown to affect tumor cell migration in 3-D environments [44].

The observation of phenotypic switching is consistent with the prediction of SVM classification (Figure 2C), which although none of the microenvironment parameters individually are sufficient predictors of tumor cell motility phenotype, a change in one or more of them in the context of the others can modulate and even switch the tumor cell behavior. Therefore, a decrease in collagen fiber density, depending on the context of other parameters (Figure S7) may transform the microenvironment that favors the invadopodial phenotype (Figure 2C, red sphere) into the microenvironment that favors fast-locomotion (Figure 2C, blue sphere), in turn switching the motility phenotype; similarly, an increase in collagen fibers may induce a switch from fast- to slow-locomotion is worth noting that a similar phenotypic switch, from MMP-dependent to MMP-independent motility, has been previously demonstrated *in vitro* by inhibiting ROCK or MMPs in melanoma cells [47],[48] and fibrosarcoma cells [49]. However, previous to our results reported here, it was unclear if such a phenotypic switch can occur *in vivo* and what collagen density would regulate this phenotypic switch.

**Figure 6:**
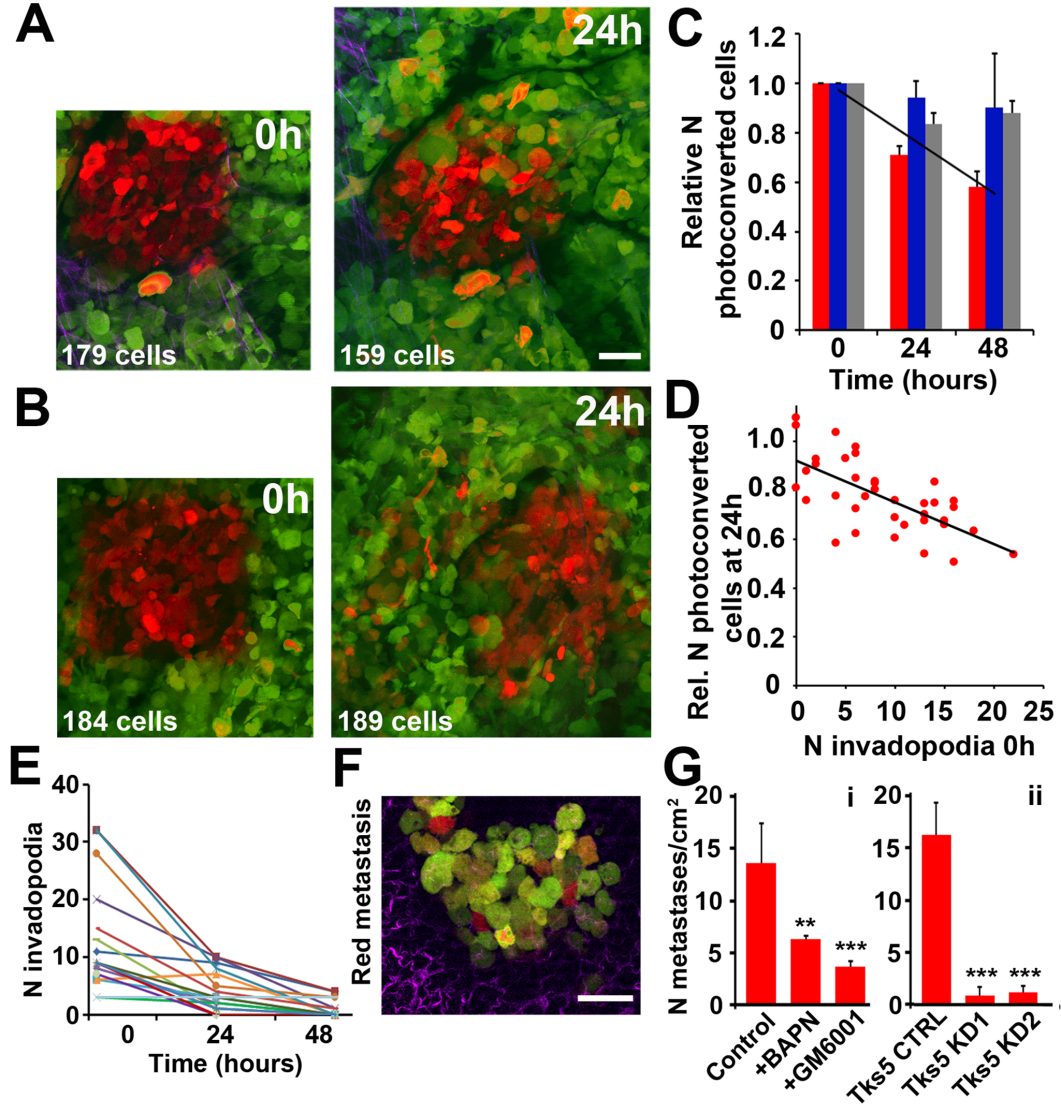
Photoconversion-enabled fate mapping links invadopodia with tumor cell dissemination and metastasis. MDA-MB-231-Dendra2 tumors were photoconverted from green to red in the areas shown and representative images are shown for the 0 and 24 times after photoconversion. (A) In microenvironments with invadopodia, photoconverted (red) cells disappeared from the field of view and their numbers are reduced, suggesting intravasation. Photoconverted cell counts are shown on the bottom left. Scale bar: 40 µm. (B) In microenvironments of fast-locomotion, photoconverted (red) cells dispersed throughout the field of view without a decrease in number. (C) Relative number of photoconverted cells measured in microenvironments where either invadopodia (red bars) or fast-locomotion (blue bars) were present at 0 h, or in animals treated daily with GM6001 (grey bars). Measurements were normalized to 0 h and corrected for cell division (see Materials and Methods). The number of photoconverted cells shows a negative trend only in fields that contain invadopodia (*p* = 1.4×10−^2^, based on linear mixed effects model). Bars are means ± SEM. (D) Negative correlation was found between the relative number of invadopodia at 0 h and the number of photoconverted cells remaining in the field at 24 h (R^2^ = 0.45, *p* = 5.4×10^−6^). (E) The number of invadopodia in the field of view significantly decreases (paired *t*-test, *p*<10^−4^) in animals treated with GM6001. Invadopodia were monitored in matched fields of view and each field is represented by different color. For (C–E), measurements were done on 37 fields in ten animals. (F) Photoconverted (red) tumor cells were traced to lungs at 5 days post-photoconversion, where some metastases were a mix of tumor cells in red (arrived at the lung at 0–5 days), orange or yellow (photoconverted cells that divided and synthesized additional green Dendra2), and green (arrival time cannot be traced). (G) The number of lung metastases containing red cells counted 5 days post-photoconversion (control) is significantly affected by BAPN or GM6001 treatment panel i. Red metastases are also largely diminished in Tks5 KD tumors compared to Tks5 CTRL (panel ii), **p<0.01, ***p<0.001 based on paired *t*-tests; based on contingency analyses, χ^2^>20 for all treatments. Average values represent sum of lung metastases across 1 cm^2^ (200 fields of view) in *n* = 3 animals per condition are shown along with ±SEM.

Finally, we hypothesized that the two motility phenotypes may lead to different tumor cell fates (Figure 6). To test this, we photoconverted subpopulations of tumor cells within microenvironments where either slow- (Figure 6A) or fast-locomotion (Figure 6B) was present. A 175×175 µm^2^ square was photoconverted within each of the four to 12 neighboring fields of view at 0 h (see Figure S6), enabling us to follow the tumor cell fate at subsequent timepoints (Materials and Methods and [6],[29] for experimental details). In the fields with invadopodia, there was a significant negative trend in the number of photoconverted (red) cells at 24–48 h (Figure 6C, red bars), previously shown to be due to dissemination from the primary tumor [6],[29],[32],[50]. In contrast, other fields that contained fast-locomoting cells had cell numbers that were mostly unchanged and sometimes increased in numbers of photoconverted cells, likely a result of the cell division (Figure 6C, blue bars) [6]. In addition, the number of invadopodia at 0 h shows very strong negative correlation (*p* = 5.4×10^−6^) with the number of photoconverted cells at 24 h (Figure 6D), suggesting that the presence of invadopodia is directly linked to tumor cell disappearance from the field of view and, possibly, tumor cell dissemination [3]. Next, GM6001 irreversibly eliminated invadopodia (Figure 6E) and inhibited the disappearance of red cells (Figure 6C, grey bars), supporting the hypothesis that tumor cells with invadopodia disseminate from the primary tumor [29].

To confirm that the disappearance of red cells in the areas rich with invadopodia (but not in areas with fast-locomoting cells) is due to the intravasation, we tested alternative processes that may contribute to changes of red tumor cell numbers, such as apoptosis and failure to proliferate (Figures S9 and S10). By combining photoconversion *in vivo* with additional *ex vivo* labeling (Figure S9), it is possible to asses rates of proliferation (Figure S9A and S9C) and apoptosis (Figure S9B and S9C) in fields of view with either fast- or slow-locomoting cells. No significant differences were observed in the proliferation rates of fields of view associated with slow- and fast-locomotion and there was practically no apoptosis observed. To confirm such an observation, an injectable marker SR-FLIVO was used for direct apoptosis measurements in real time (Figure S10), combined with cisplatin treatment to induce apoptosis in the positive control group. This method also reported the absence of apoptosis in areas where fast- or slow- locomotion occurs, strengthening the link between the red cells disappearance and intravasation. Following disseminated red cells to the secondary organs, we monitored the number of tumor cells that had formed lung metastases at 5 days post-photoconversion (Figure 6F and 6G). Some lung metastases contained red tumor cells (photoconverted cells that arrived at the lung at 0–5 days from the photoconversion site in the primary tumor), orange and yellow tumor cells (photoconverted cells where red/green Dendra2 ratio is reduced due to the cell division and synthesis of new green Dendra2), and green tumor cells (whose origin cannot be traced) (Figure 6F). When tumors were chemically treated with BAPN (which selectively inhibits invadopodium formation by modifying the external microenvironment) or GM6001 (which inhibits ECM degradation by invadopodia) starting at photoconversion time (0 h, see Materials and Methods), we observed a significant reduction in the number of metastases containing red tumor cells (Figure 6G, panel i), pointing to onset of inhibition of dissemination at 0 h. Moreover, lung metastases were practically eliminated in Tks5-knockdown tumors (Figure 6G, panel ii) where invadopodia were removed, while the fast-locomotion phenotype remained largely unaffected (Figure S4G). These data together support the hypotheses that invadopodia and ECM degradation by tumor cells are essential for dissemination and metastasis, and that metastasis does not occur without active ECM degradation by tumor cells.

## Discussion

In this study, we monitor two different phenotypes of tumor cell motility: fast-locomotion and invadopodium-associated slow-locomotion, which appear in spatially separate microenvironments in primary, orthotopic xenograft tumors. Such a separation of phenotypes among different fields was striking and suggests that the size of the imaging field is well below the average size of each phenotypic microenvironment. As both phenotypes can be seen in the same animal (imaging window covers ∼50 mm^2^) but they did not commonly occur in the same (∼0.5 mm^2^) or adjacent fields, we estimate the phenotypic microenvironment size to be 1–50 mm^2^.

Although both motility phenotypes are dependent on the presence of vasculature, ECM, and macrophages, they do not show obvious dependency on any of these parameters, either individually or when (linearly) combined. However, by using a multiparametric, non-linear SVM classification, we were able to predictably classify microenvironmental parameters that favor each motility phenotype. The non-linear nature of this statistical model exposes the complexity of tumor microenvironment, a theme often addressed in reviews, but commonly avoided in experimental science, where the focus is on individual microenvironmental parameters and regard others as stable and homogeneous “background”. Here we show that more than one microenvironmental component is involved in phenotype determination in primary tumors and also, that small regions with genetically identical tumor cells may exhibit different phenotypes. We have analyzed five microenvironment parameters (number of macrophages, density of collagen fibers and tumor cells, number and size of blood vessels), which all contribute to the determination of tumor cell motility phenotype to a smaller or larger extent. The extent of contribution of each parameter depends on the context of the other four parameters (Figure S7) and can change through time. Hence, heterogeneity, complexity, and dynamical changes in the tumor microenvironment dominate tumor cell phenotype and should be considered in the interpretation of future intravital imaging studies and in the development of new diagnostics and treatments. Stepping away from the focus on single microenvironment parameter analysis towards the multiparametric analysis may not only contribute to generating a more comprehensive view of how tumor microenvironments determine cell behavior, but also help with the search for new therapeutic targets, which is currently conducted by analysis of individual molecules or specific protein interactions.

SVM classification suggests the existence of a fine balance among chemical and mechanical signals produced by tumor cells, macrophages, blood vessels, and ECM, which determines the motility phenotype of tumor cells. Ongoing changes in one or more microenvironment parameters (collagen fibrillogenesis, angiogenesis, macrophage recruitment, etc.) can lead to a switch between fast-locomotion and invadopodium-associated slow-locomotion. It is likely that both motility phenotypes contribute to metastasis. Fast-locomotion is more efficient in translocation of tumor cells, which may transport them to regions where they stop due to ECM composition and stiffness, such as in regions adjacent to some blood vessels as shown here, and then shift to the invadopodium-associated phenotype that results in tumor cell dissemination. The presence of misclassifications (Figure 2C, green dots) on the borders between two phenotypes suggests that there may be microenvironmental conditions where the switch between motility phenotypes commonly takes place. In further support of this, we were able to modulate invadopodium frequency by changing one of the parameters, collagen fiber density (Figure S6). A decrease in collagen fiber density resulted in elimination of invadopodia and, in some areas, a switch to fast-locomotion. In theory, changing other parameters such as blood vessel size and macrophage number would achieve a similar result (Figure S6). To understand the mechanism of this phenotypic switching, further mathematical modeling and direct *in vitro* measurements are currently underway. We speculate that both motility phenotypes are likely to be exhibited by the same tumor cells and that there are no genetic mutations unique to the two motility phenotypes. In support of this, we see both phenotypes in a xenograft mouse model, based on genetically identical tumor cells. Interestingly, the fraction of cells exhibiting either of the phenotypes in a particular field of view is the same, 15%. Similar fractions of tumor cells were previously reported as capable of assembling invadopodia *in situ*, tumor sections, or *in vitro*, when plated on thin gelatin [29]. This leads to the suggestion that both fast- and slow-locomotion are phenotypes that arise when similar internal conditions are present (motility cycle is active) but the cell is exposed to different external conditions. We propose that within the same region, under the same external conditions, the other 85% cells are not capable for motility at the moment, and the reason for that may be that they are mitotic or bound by energetic and signaling constrains. Broadening this hypothesis into other motility phenotypes, similar fractions of cells were also reported in individually or collectively migrating cells in mammary carcinoma xenografts, where phenotype was dependent on intracellular transforming growth factor beta (TGFβ) expression [51].

We demonstrated that the slow-locomotion phenotype, which was not previously characterized *in vivo*, is associated with small tumor cell protrusions identified as invadopodia using morphological, molecular, and functional assays. This motility phenotype is linked to ECM degradation, tumor cell dissemination from the primary tumor and subsequent lung metastasis, and inhibited by MMP-inhibitor. It is worth noting that the treatment with GM6001 was previously shown in 2-D culture to minimally affect existing invadopodial core structures [52], but their growth and extension were not tested. In fact, invadopodia in 3-D grow in cycles of extension-retraction [20] and degradation of ECM by MMPs followed by enlargement or elongation of invadopodia was suggested to be a part of a positive feedback mechanism [37]. We propose that by abolishing this cycle, GM6001 inhibits elongation, rendering protrusions too small to be detected *in vivo*. In summary, our results support the hypothesis of a positive feedback loop between products of MMP proteolysis and continued invadopodial extension [37]. Based on the evidence collected *in vitro* in 2-D and 3-D [12],[15],[53], we hypothesize that membrane-tethered MT1-MMP1 is likely enriched in the actively degrading invadopodia compared to fast-locomoting cells. However, none of the available antibodies were able to give us a satisfactory signal. While experimentally, MMPs remained only broadly tested using GM6001, we speculate that the key role of MMPs includes immobilization of MT1-MMP on invadopodia, leading to local activation of all MMPs present in the external microenvironment [54].

## Materials and Methods

### Ethics Statement

All procedures involving animals were conducted in accordance with NIH regulations, and approved by the Albert Einstein College of Medicine IACUC, by 20101010 and 20130909 protocol numbers.

### Cell Lines and Animal Models

A metastatic human breast cancer line MDA-MB-231 was cultured and maintained in DMEM media supplemented with 10% fetal bovine serum (FBS) and 50 U penicillin/50 µg streptomycin per ml. The Dendra2-MDA-MB-231 cell line [50] was created by electroporation of the Dendra2 cloning vector C1 with the geneticin selection marker [6]. We performed fluorescence-activated cell sorting (FACS) after 14–20 days of selection; after removing the highest-expressing top 5%, we kept the top 20% of the highest fluorescing cells and maintained them under selection using 500 µg/ml geneticin (Invitrogen). No changes in cell morphology, viability, or proliferation were observed in the labeled MDA-MB-231 cells compared to the parental cell line. Cell lines labeled with cortactin-GFP [20] and cortactin-TagRFP [19] have been previously described. Tks5 knockdown cell lines Tks5 KD1 and Tks5 KD2 and knockdown control cell line Tks5 CTRL were all created by transduction of Dendra2-MDA-MB-231 cells with lentiviral particles (five particles/cell), which contained shRNA in pLKO.1 vector, targeting Tks5 (knockdowns) or non-targeting shRNA (CTRL) (Sigma Aldrich MISSION library based on Broad Institute consortium). Tumors were formed by injecting 10^6^ cells in 150 µl of 20% collagen I in PBS into the mammary fat pads of 5–7 week old SCID mice. Imaging experiments were done 9–12 weeks after injection.

### Multiphoton Microscopy

Multiphoton imaging was done on a custom-built system based on an inverted Olympus IX71 microscope and two Ti-Sapphire lasers, one of them equipped with an Optical Parametric Oscillator (OPO) extension [55]. The first laser was tuned to 880 nm for excitation of GFP-like fluorophores and Texas Red, while the second laser was tuned for photoconverted Dendra2 at 1035 nm and MMPSense 680 at 1250 nm [55]. For experiments involving simultaneous Dendra2 and MMPSense 680 imaging, MMPSense680 was excited using the 880 nm laser. The microscope system is equipped with four simultaneously acquiring detectors, which allowed simultaneous imaging of collagen fibers via second harmonic generation (blue) and fluorescence from GFP-like fluorophores (green), Texas Red or photoconverted Dendra 2 (red), and MMPSense (far red).

In the photoconversion-enabled fate mapping experiments, a commercially available microscope system with multiphoton and confocal modes (Olympus FV1000MPE with ULTRA 25×, 1.05 NA water immersion objective) was used for initial 4-D imaging over 30 min (in multiphoton mode, with laser locked at 880 nm) and subsequent photoconversion of Dendra2 (in confocal mode, using 405-, 488-, and 546-nm laser lines).

Stacks (512 µm×512 µm×100 µm×30 min) were collected at 5-µm-thick z-sections at 3-min timepoints for time-resolved series. Imaging was done at 0.25 µm^2^/pixel.

### Continuous Imaging Sessions Using Skin Flap

Multiphoton imaging of mammary tumors was done as described previously [56]. MDA-MB-231 cells labeled with Dendra2- or cortactin-GFP were injected into the mammary fat pads of 5–7 week old SCID mice. After 10–12 weeks, we injected Texas Red 70 kDa dextran (250 nmol in 100 µl PBS per injection) [5] for macrophage labeling and performed skin flap surgery 2 h later on anesthetized animals. Exposed mammary tumors were positioned on top of a coverslip on an inverted microscope and imaged continuously using a custom-built two-laser multiphoton microscope for up to 3–5 h. In some experiments, additional tail-vein injections were done for blood vessel labeling using Texas Red 70 kDa dextran (Invitrogen; 250 nmol in 100 µl PBS per injection), MMPSense 680 (Perkin Elmer; 2 nmol in 150 µl PBS per injection), SR-FLIVO (Immunochemistry Technologies; 1∶10 dilution, 2 h prior to imaging), or the pan-MMP inhibitor GM6001 (Milipore; 1 µmol in 100 µl injection). A stock solution of GM6001, 500 mM in DMSO, was diluted in sterile PBS before tail vein injection. Post-surgical injection assures that compounds are only present in tumor blood vessels that are intact, connected to tumor vasculature and flowing at the time of injection.

### Photoconversion-Enabled Tumor Cell Fate Mapping through the Mammary Imaging Window

As previously described [6],[29],[50],[57]–[59], the mammary imaging window was combined with photoconvertible Dendra2 as a cytoplasmic marker. Briefly, Dendra2-MDA-MB-231 cells were injected into the mammary fat pads of 5–7 week old SCID mice. After 9–11 weeks, a mammary imaging window was implanted on top of the growing tumor (5–7 mm in diameter). Following a 3-day recovery, mice were anesthetized and placed in the imaging chamber on an Olympus FV1000-MPE hybrid multiphoton-confocal system. In each area, ten z-sections were acquired (total stack size is 512 µm×512 µm×100 µm) over 30 min. One to three areas were imaged per animal. Subsequently, photoconversion was done in 175×175 µm areas, separated by 150 µm along both x and y axes (Figure S6). Photoconversion areas were scanned for ten to 20 cycles with a 405-nm laser in confocal mode. Further imaging was done using the two-laser multiphoton system, using 880 nm for imaging of green species of Dendra2 and 1,035 nm for red species of Dendra2. 3-D stacks were collected at photoconverted sites at 0 h, 24 h, and 48 h post-photoconversion. The number of red (photoconverted) Dendra2 cells was counted from the RGB overlay on individual z-sections, using manual Cell Counter plugin in ImageJ. Reported numbers are sums of counts at five z-sections separated by 25 µm, for method details see [29]. Values were corrected for cell division by dividing by control values from areas where no motility was detected [6]. The dispersion of photoconverted cells is monitored as the surface embedding all red cells in the 0–100 µm maximum image projection [6].

In MMPSense 680-based degradation measurements (Figure 4B–4D), in SR-FLIVO apoptosis measurements (Figure S9), and in microenvironment modulation experiments (Figure 5), photoconversion was used to locate the same microenvironments at 0–48 h. Intravenous injections of 150 µl MMPSense 680 were done at 0 h, intraperitoneal injections of 150 µl PBS containing either 1 g/kg/day L-ribose or 20 mg/kg/day BAPN were done at 0 h and 24 h, while intraperitoneal injections of 2 mg/kg/day cisplatin were done daily for 5 days.

### Photoconversion-Enabled Tumor Cell Fate Mapping in Pulmonary Metastases

As previously described [55], transdermal photoconversion of Dendra2 can be used for fate mapping in pulmonary metastases. Briefly, 12 weeks after injection of Dendra2-MDA-MB-231 cells, pairs of SCID mice were matched by tumor size from the same cage. In each pair, one mouse was injected intraperitoneally with 150 µl of sterile PBS (control), while the other was injected with 1 µg GM6001 in 150 µl PBS (GM6001-treated [31]). Six hours later, mice were anesthetized by isoflurane and hair was shaved off the top of the mammary tumor. The tumors were photoconverted transdermally, using a 405-nm LED photodiode array [55]. Treatments with PBS or GM6001 were repeated daily, and mice were sacrificed at 5 days. Lungs were taken out and metastatic colonies (groups containing 1+ tumor cells) were counted in 50 fields of view through the ocular of the microscope, using a 25× objective. Colonies containing 1+ red tumor cells were classified as red, although most also contained green cells owing to the cell division over 5 days. Representative images (Figure 6F and 6G) were taken using an Olympus multiphoton setup, using the same imaging parameters as for intravital imaging. Counted colonies were analyzed by contingency table and compared for their average values (Figure 6H), resulting in significant differences between control and GM6001-treated red metastases (χ^2^ = 20.04, *p* = 0.048).

### Immunofluorescence and Immunohistofluorescence

Labeling of invadopodia in cell culture was done as previously described [19],[20],[29]. Briefly, 50 k MDA-MB-231-Dendra2 cells (Tks5 CTRL, Tks5 KD1, and Tks5 KD2) were plated in triplicate for 6 h on Mattek dishes coated with Alexa405 gelatin. After this time, they were fixed for 15 min in 3.7% paraformaldehyde (PFA), washed three times in PBS, and permeabilized for 5 min in 0.1% Triton X-100, blocked for 2 h in 1% BSA and 1% FBS, and incubated for 1 h in primary antibodies against cortactin (Abcam, ab33333) and Tks5 (Santa Cruz, M-300) and 1 h in secondary Alexa antibodies.

Labeling of invadopodia in tissue sections was done as previously described [29]. MDA-MB-231 tumors (either -cortactin-GFP or -Dendra2) were excised and fixed overnight in 3.7% PFA, washed for 1 h in cold PBS and dehydrated overnight in 30% sucrose. They were embedded in Optimal Cutting Temperature (OCT) compound, cut to 6-µm sections, permeabilized for 10 min with 0.1% Triton X-100, blocked for 2 h in 1% BSA and 1% FBS, and finally incubated for 2 h with primary antibodies against degraded collagen (Ibex Pharmaceuticals, Col2¾C short antibody, 1∶100 or immunoGlobe Col1-3/4C, 1∶100) and Tks5 (Sigma, mouse anti-SH3PXD2A, 1∶100). Secondary antibodies were conjugated to AlexaFluor-543 (Invitrogen, 1∶300) and AlexaFluor-633 (Invitrogen, 1∶300) and mixed with DAPI (Invitrogen, 1∶1,000). Alternatively, sections were permeabilized for 10 min in cold acetone and incubated with primary antibodies against endomucin (V.7C7, Santa Cruz Biotechnologies, 1∶200) as well as tumor ECM components such as laminin (ab11575, Abcam, 1∶100), collagen IV (2150-1470, AbD Serotec, 1∶100), collagen I (ab90395, Abcam, 1∶100), or fibronectin (ab6328, Abcam, 1∶100). Secondary antibodies were conjugated to AlexaFluor-488 or -543 (Invitrogen, 1∶300). Cells were imaged with a Leica SP5 confocal microscope (Figures S3 and S4).

Labeling of tumor cell proliferation and apoptosis post-photoconversion was done by cryosectioning of the primary tumor regions under the mammary imaging window (top 150 µm, [60]). Neighboring sections were labeled by Ki67 (DAKO) and cleaved caspase 3 (Milipore), using Alexa633 as secondary antibodies.

### Blood Vessel Coverage by ECM Components

Blood vessel coverage by ECM components, collagen I and IV, laminin and fibronectin was measured using ImageJ (Figure S2). Briefly, images of blood vessels and ECM proteins were thresholded and tested for colocalization using Manders' M2 coefficient [61]. Blood vessel edges were traced using the green channel, with a 5-µm-thick band generated to encapsulate the area of ECM proteins. The amount of ECM components deposited around blood vessels was measured and normalized to the surface area.

### MMPSense 680 Validation

MMPSense 680 (Perkin Elmer) is a polymer with quenched VT680 chromophore and is weakly fluorescent prior to exposure to MMPs. Proteolytic cleavage of the peptide that links the copolymer probe can be executed by a wide range of MMPs and produces a signal in the near-infrared range (with emission peak at 680 nm) [62]. As MMPSense 680 is soluble, it can be added either to culture media or injected directly into the animal vasculature.

To validate MMPSense 680 as a reporter of MMP-driven proteolytic activity at single cell resolution (Figure S5), a comparison was made to an established marker of matrix degradation, FITC-DQ-collagen I (Invitrogen) in 3-D culture based on rat-tail collagen I (BD Biosciences). A 2–4-µm-thick layer of DQ-collagen I (10 µg/ml) and collagen I (2 mg/ml) mixture (1∶100, DQ-unlabeled collagen) in cold, phenylalanine-free DMEM/10% FBS serum was established on the bottom of a Mattek dish. After 20 min at 37°C, 5×10^4^ MDA-MB-231-cortactin-TagRFP cells were seeded on this layer and covered with additional collagen I. After 60 min at 37°C, we added 50 nM MMPSense 680 and cultured the cells for 6–24 h. Image stacks were collected at 0, 6, and 24 h using a Leica SP5 confocal microscope, utilizing 488-, 543-, and 633-nm laser lines and setting the prism for detection at 500–525, 555–610, and 670–740 nm. Image processing and quantification of the amount of degraded ECM, measured as percent of total image area after threshold, was done in ImageJ. Briefly, 3-D stacks of degraded DQ collagen or MMPSense 680 were combined into a maximum intensity projection and processed using a 1-pixel median filter. Thresholding of images taken at 24 h (Figure S5A) was done using average values of images taken at 0 h.

### Intravital Systems Microscopy

The workflow of our intravital systems microscopy approach is outlined in Figure 2A and 2B. Each 4-D image stack was collected in three or four channels. After a brief quality screening of each z-section, two adjacent z-sections were combined into a maximum intensity projection (10 µm thick) and the recorded stack was separated into four neighboring fields of view (256×256 µm) for easier processing. All images were passed through smoothing filters and thresholded to remove background fluorescence. Individual microenvironment time lapses were passed through the StackReg/TurboReg plugin [63], available at http://bigwww.epfl.ch/thevenaz/stackreg/. These Java classes efficiently remove minor drifts in the xy plane resulting from breathing and tissue settling.

Time-lapse movies of individual z-sections in each field of view (30′–60′ duration) were first visually scored for morphological determinants of tumor cell fast locomotion, as previously described [4]–[6],[29],[31]–[34]. Briefly, the fast locomoting cell translocates by the extension of the cell front, movement of the center and the contraction of the rear, with an average speed of 1 µm [34]. To account for the number of fast locomoting cells the frame taken at 0′ was subtracted from the frame taken at 30′, both in the green channel, yielding an image of all pixels translocated during this time interval. Particle analysis and Region of Interest (ROI) Manager in ImageJ were then used to count number of locomoting cells and overlay them with the original 0′ frame to show initial tumor cell position and the trace left by the tumor cell migration over 30′ (Figure 1B, green for tumor cells and blue for the tumor cell tracks). Some tumor cells moved out of the analyzed z-section during the 30′ period and were traced in the neighboring z-sections. In such cases, cell tracks were visualized via maximum projection, accounting for the thickness of the z-slice (5 µm).

Next, time-lapse movies were viewed at 2–4× zoom, exposing small protrusions characterized by (a) finger-like morphology, (b) width of 1–3 µm, and (c) dynamics. Frame subtraction and particle analysis were used to visualize the dynamics of small protrusions over 30′ (tip movement and change in size due to the cycles of extension and retraction) as well as the number of small protrusions per cell (Figures 1F, red line, and 2D). In order to detect migration of the tumor cell body (front, center, and rear) in cells with small protrusions, movement of time-lapse movies were extended to 5 h. The following five microenvironment parameters were extracted from the multichannel 3-D stacks: (a) density of collagen fibers, defined as percent area above threshold in the blue channel, (b) density of tumor cells, defined as percent area in the green channel, (c) number of macrophages, defined as macrophages labeled with 70 kDa dextran [5], (d) number of flowing blood vessels in the field of view, and (e) diameter of the largest flowing blood vessel visible in the field of view. For (d) and (e), we made the assumption that all blood vessels flowing at the time of post-surgical injection by Texas Red were labeled. As macrophages and blood vessels were collected in the red channel, prior to measurements they were separated on the basis of size and morphology, using the Analyze Particles plugin in ImageJ.

Microenvironment parameters and the motility phenotype (i.e., fast- or slow-locomotion) form a multiparametric matrix that we used for SVM classification [64]. Different types of kernels and other parameters were tested systematically in R software. Classification was finally done using Gaussian radial basis kernel, using the “tune.svm” procedure in the R-package “e1071,” starting from the default γ- and cost-parameters and iteratively optimizing them to increase accuracy, which we tested using 10-fold cross-validation. The dataset was divided into randomly grouped ten equal-size sets, training on nine while testing on the left-out set. Starting from SVM based on three-parameter combinations, we were able to achieve 78%–84% accuracy (Figure S1D). By adding all five parameters measured, we ultimately achieved 92% accuracy. Without cross-validation (i.e., using all data) classification accuracy is 95%. This means that we were able to predict motility phenotype on the basis of measurements of all five microenvironment parameters in 92/100 fields of view and that removing any of the parameters reduces the predictive power of SVM. However, for purposes of presentation in 3-D space, we have used a 3-D projection that contains parameters with the highest influence: density of collagen fibers, number of macrophages, and diameter of blood vessels (Figure 2C). Misclassifications (Figure 2C, 5/100, green dots) are present in boundary areas. Note that blue- and red-shaded areas in Figure 2C are provided only as an illustration.

### Data Processing and Analysis

Collected image stacks were processed by a combination of the ImageJ [65] and Imaris software packages. ImageJ was supplemented with custom-written plugins based on our work and work of others for image browsing, color correction, and migration trajectories. We used Imaris 7.4 for 3-D reconstructions. Data was directly exported to Microsoft Excel for statistical analysis and plotting; the R package was used for trend analyses, variance trend analysis, SVM classifications, correlations, linear regression, and calculation of *p*-values, while Matlab was used for SVM representation. All numerical values used in figure graphs are included in Data S1 file.

### Variance Trend Analysis

To test the influence of the blood vessel distance and diameter on tumor cell motility phenotypes (Figure 3B), we measured the shortest distance of the tumor cell front to the nearest blood vessel. We hypothesized that in case the blood vessel is the source of a chemical or mechanical gradient, then the increase in blood vessel diameter will allow motility at larger distances, thus increasing the spread of distances (variance) of motile tumor cells from the blood vessel. To test this hypothesis, we first calculated the residuals that result from regressing the observed distance of the cell front on the vessel diameter. In turn, the log square residuals were regressed on the vessel diameter. As hypothesized, an increase in blood vessel diameter was associated with a significant increase in residuals in cells with small protrusions (R^2^ = 0.3, p = 7×10^−8^) indicating that in the fields of view where only microvessels are present, small protrusions form only next to the blood vessel, while in the presence of large-diameter blood vessels, small protrusions can be both close and far from the blood vessel. Fast-locomoting cells exhibit no such behavior (R^2^ = 0.07, *p* = 10^−3^). Note the small R^2^ value. Red-shaded area in Figure 3B illustrates variance trend as blood vessel diameter increases.

## Supporting Information

Figure S1 **Distinguishing between tumor microenvironment conditions where fast- or slow-locomotion occurs can not be done with linear models but with non-linear SVM modeling.** (A) Model selection using any of 1, 2, 3, 4, or 5 microenvironment parameters does not provide separability of microenvironments in which fast locomotion (blue) or slow-locomotion (red) occur. As an illustration, all 2-parameter scatter plots are shown. There are no lines that can be drawn to separate blue and red points without significant misclassification. N MF, Number of macrophages; N BV, Number of blood vessels; D BV, diameter of blood vessels; % ECM, area covered by collagen fibers; % TC, area covered by tumor cells. (B, C) Partial correlation or causal modeling do not yield statistically significant results. (D) Non-linear classification using SVM methodology resulted in 92% accuracy, with only a few misclassified points. Accuracy is based on 10-fold cross-validation. Presented here are two SVM classifications based on three out of the five microenvironmental parameters. On the left is a classification based on D BV, N MF, and % ECM, yielding 84% accuracy; on the right, classification is based on N BV,% ECM, and % TC, yielding 78% accuracy. Misclassified data (green points) are tumor cells whose observed class of protrusion is opposite to that predicted by the SVM classification algorithm. Numerical values used for analysis are the same as in Figure 2C.
(TIF)

Figure S2 **Correlation of blood vessel diameter and blood vessel coverage with ECM components.** (A–D) Blood vessels were visualized by endomucin (green) and coverage of the blood vessel was measured using laminin (red) (A), collagen I (B), fibronectin (C), and collagen I (D). Scale bar: 50 µm. (E–H) We subsequently plotted blood vessel diameter versus coverage by ECM components. While the coverage of basement membrane proteins increases linearly with blood vessel diameter (E, F), fibronectin and collagen I levels are not linked to the size of blood vessels and are present in both the perivascular and interstitial ECM (G, H).
(TIF)

Figure S3 **Cortactin is enriched at the tip of collagen-degrading protrusions in 3-D culture and tissue sections.** (A) In 3-D culture of MDA-MB-231-cortactin-GFP cells, cortactin (green) is enriched at the tip of the small protrusion, which is surrounded by degraded collagen (red, Col 2 ¾ C antibody). Cell nucleus is labeled by DAPI (blue). Scale bar: 5 µm. (B) In tissue cryosections of MDA-MB-231-cortactin-GFP tumors, cortactin is similarly enriched at the tips of small protrusions in perivascular tumor cells and degraded collagen surrounds cortactin enrichment. Scale bar: 10 µm. Arrows point to tips of protrusions. (C) Line-scans measuring cortactin-GFP along yellow lines in (A and B). Lines go through protrusions and show peaks of cortactin concentration at the tips.
(TIF)

Figure S4 **Tks5 knockdown inhibits appearance of invadopodia both** ***in vitro*** **and in the primary tumor.** (A) Western blot showing Tks5 levels in control plKO.1 cell line transduced with non-targeted shRNA and Tks5 knockdown cell lines. Knockdown efficiency is 96% and 99% for Tks5 KD1 and Tks5 KD2, respectively. (B) Representative images of Tks5 CTRL cells plated on fluorescent gelatin and labeled with Tks5 (first panel) and cortactin (second panel) antibodies. Color panel shows overlay of Tks5 (red) and cortactin (green); insert zooms in to an invadopodium *in vitro* (arrow). Right panel shows corresponding holes in fluorescent gelatin (white), created by invadopodia (arrow). Bar 5 µm. (C) Quantification of images shown in (B). Numbers of Tks5 dots (red bars) and total area of gelatin degraded over 6 h (black bars) in the control and knockdown cell lines. Bars represent means ± SEM of *n* = 4 dishes, ran as duplicates, each with ten to 15 fields of view. (D) Western blot showing levels of Tks5 in tumor cells sorted from the primary tumor. Knockdown efficiency is 89% and 95% for Tks5 KD1 and Tks5 KD2, respectively. (E) Representative cryosection of Tks5 CTRL primary tumor with Tks5 (left insert) and degraded collagen I (middle insert) antibodies. Color panel shows overlay of Tks5 (purple) and degraded collagen I (cyan); color insert zooms in to an invadopodium *ex vivo* (arrow). Right panel shows degraded collagen I (cyan), created by invadopodia (arrow). Bar 20 µm. (F) Quantification of images shown in (E). Numbers of Tks5 dots (purple bars) and total area of degraded collagen I (cyan bars) in the control and knockdown cell lines. Bars represent means ± SEM of ten to 15 fields of view. (G) The fraction of tumor cells within the same field of view which exhibit small protrusions *in vivo* and associated slow-locomotion (red, panel i) is dramatically reduced in Tks5 knockdown tumors compared to Tks5 CTRL tumors. In the same tumors, fractions of tumor cells exhibiting fast-locomotion (blue, panel ii) are either slightly affected (Tks5 KD1) or not affected by the knockdown (Tks5 KD2). Bars represent means ± SEM of *n* = 4–7 animals, each with two to six fields of view. Comparisons in (C), (F), and (G) were done using homoscedastic *t*-tests for Tks5CTRL versus KD1 or KD2 *p<0.05, **p<0.01, ***p<0.001.
(TIF)

Figure S5 **Comparison of localization and signal trends of the MMPSense 680 fluorescent reporter of ECM remodeling and degradation with established reporter DQ collagen I in 3-D culture.** (A) Raw (left) and thresholded images (right) of DQ collagen I (red) and MMPSense 680 (cyan) fluorescent signals at 6 h after addition into MDA-MB-231-cortactin-TagRFP (green) in 3-D collagen. DQ collagen I gel was mixed with collagen I 1∶10 and MMP Sense 680 solution was mixed with culture media 1∶100. Optimal threshold levels were determined by averaging images taken at 0 h (Materials and Methods). Maximum projection of 5 µm total is shown. Scale bar: 15 µm. (B) Overlay image of DQ-collagen I, MMPSense 680, and tumor cells shows that DQ-collagen I and MMPSense 680 signals have similar localization, surrounding motile cells, with MMPSense 680 signal being more diffuse. (C) Fluorescence above threshold in the control gel or in the presence of tumor cells with or without treatment with 25 µM GM6001. Fluorescent signal indicating degraded ECM was significantly increased in the presence of tumor cells, but was reduced when those cells were treated with GM6001. The same trends were observed with both DQ collagen I and MMPSense 680. **p<0.01, ***p<0.001, using homoscedastic *t*-test. Bars represent means of 10–15 fields of view ± SEM; *n* dishes = 3, done on separate days.
(TIF)

Figure S6 **Photoconversion-enabled tumor cell fate-mapping experiment designs.** (A) Photoconversion illustration: mammary imaging window implanted on top of the primary tumor contains a round glass coverslip 8 mm in diameter. Four to 12 512×512×100 µm stacks are recorded within the same animal, each one containing a 175×175 µm photoconverted region. (B) Tumor cell fate-mapping in four tiled regions: photoconverted cells (red), non-photoconverted cells (green), and collagen (purple) are monitored at 0, 24, and 48 h. To detect all red cells that dispersed from the original region over time, visualized areas at 24 h and 48 h are increased by recording four separate stacks and stitching them into a mosaic stack. Images shown are maximum projections of ten sections combined to 50 µm in depth. Scale bar: 50 µm. (C) Photoconversion followed by MMPsense 680 injection: tumor cells are photoconverted at 0 h (top panel), followed by MMPSense 680 tail-vein injection (MMPSense 680, cyan; collagen was excluded for clarity), which extravasates and accumulates in perivascular regions, locating spots of ECM degradation. Maximum projections of total 50 µm in depth are shown. Graph shows how normalized MMPSense 680 signal increases over time and plateaus at 24 h (see Materials and Methods).
(TIF)

Figure S7 **Illustration of context dependency principle.** A shift from level 2X to level 1X in the parameter X will cause a phenotypic switch from slow-locomotion (red) to fast locomotion (blue) motility when parameter Y is at level 1Y, but not when parameter Y is at level 2Y. In the experiment shown in Figure 5, parameter X is collagen fiber density, which we manipulated by changing ECM cross-linking using ribose or BAPN. Parameter Y may be any of the other four parameters (number of macrophages or blood vessels, blood vessel diameter, or tumor cell crowding).
(TIF)

Figure S8 **Collagen fiber density** ***in vivo*** **modulation by treatment with L-ribose or BAPN.** (A) Collagen fibers imaged in the same field of view at 0, 24, and 48 h. (B) Collagen fibers imaged 0–48 h in an animal treated with 1 g/kg/day L-ribose. (C) Collagen fibers imaged 0–48 h in an animal treated with 6 mg/kg/day lysil oxidase (LOX) inhibitor BAPN. Scale bar: 75 µm.
(TIF)

Figure S9 **Proliferation and apoptosis rates are similar in fast- and slow-locomoting photoconverted cells.** (A) Proliferation in areas of fast- and slow-locomotion: Representative image of tumor cells in photoconverted area visualized via multiphoton microscopy *in vivo* at 24 h (green, non-photoconverted cells; red, photoconverted cells, left panel); *ex vivo*, same area visualized via confocal microscopy after cryopreservation and staining with Ki67 (cyan, middle panel) and *ex vivo*, same area when Ki67 and tumor cells are overlayed (right panel). (B) Apoptosis in areas of fast- and slow-locomotion: Representative image of tumor cells in photoconverted area visualized via multiphoton microscopy *in vivo* at 24 h (green, non-photoconverted cells; red, photoconverted cells, left panel); *ex vivo*, same area visualized via confocal microscopy after cryopreservation and staining with caspase 3-cleaved (purple, middle panel) and *ex vivo*, same area when caspase 3-cleaved and tumor cells are overlayed (right panel). Scale bar 40 µm. (C) Numbers of proliferating and apoptotic cells (practically none) in the photoconverted areas where fast- (blue bars) or slow-locomotion (red bars) were detected at 0 h.
(TIF)

Figure S10 **Apoptosis in areas of fast- and slow-locomotion measured directly using SR-FLIVO marker.** (A) SR-FLIVO marker mixed in culture media conditions does not label any cells, but in the positive control treated by 50 µM cisplatin for 2 h, it concentrates in apoptotic cells. Tumor cells (green, left), SR-FLIVO in media (red, middle), overlay (right). Bar 20 µm. (B, C) SR-FLIVO marker *in vivo* does not label any cells in fast- or slow-locomotion areas (B), but concentrates in apoptotic cells induced by 5 day-treatment with 2 mg/kg cisplatin (C). Tumor cells (green, left), SR-FLIVO *in vivo* (red, middle), overlay (right). Bars 80 µm. (D) Numbers of apoptotic cells in the areas of fast- (blue bars) or slow-locomotion (red bars) were detected or in the animals treated with cisplatin (grey bars).**p<0.01.
(TIF)

Figure S11 **Principle of support vector machine classification.** (A) Non-linear transformation of input space increases dimensionality and allows for linear separation. (B) Classification using support vectors to find optimum hyperplane (Z).
(TIF)

Data S1 **Numerical data used to generate plots in** **Figures 1** **–** **6** **and S1, S2, S3, S4, S5, S6, S7, S8, S9, S10, S11.**
(XLSX)

Movie S1 **Representative examples (related to** **Figure 1** **) of areas of tumor cell fast locomotion (panel a, related to** **Figure 1A** **) or slow-locomotion (panel b, related to** **Figure 1D** **).** 30′ time lapses.
(MOV)

Movie S2 **Representative examples of small protrusions (related to** **Figure 1E** **).** Examples of small protrusions directed towards collagen fibers (panels a, b) or blood vessels (panels c, d). Some small protrusions in direct contact with blood vessel are protruding into the blood vessel (panels c, d). 30′ time lapses.
(MOV)

Movie S3 **180 degree rotation of 3-D plot shown in** **Figure 2C** **.** Rotation is done around axis Number of macrophages (MF), while the other two axes are diameter of blood vessels (Dmax) and percent collagen fibers (%Col). Blue balls represent microenvironments that enable fast locomotion and red balls those enabling slow-locomotion.
(MOV)

Movie S4 **Representative example (related to** **Figure 4A** **) of homogeneous cortactin levels in tumor cells during fast locomotion (panel a) and cortactin enrichment in the small protrusions (panel b).** 30′ time lapses.
(MOV)

Movie S5 **In MDA-MB-231-cortactin-GFP tumors (related to** **Figure 4B** **), MMPSense 680 injected into the circulation extravasates and colocalizes with cortactin-enriched protrusions (panel a), but not with cell front in the fast locomoting cell (panel b).** 3h time lapse. Several images were omitted due to breathing artifacts.
(MOV)

## Abbreviations

BAPN: β-aminopropionitrile
FBS: fetal bovine serum
KD: knockdown
ECM: extracellular matrix
MMP: metalloprotease
SVM: support vector machine
