# Supplementary figures and images for: Multiparametric Classification Links Tumor Microenvironments with Tumor Cell Phenotype
Source: PLoS Biol. 2014 Nov 11;12(11):e1001995. doi: 10.1371/journal.pbio.1001995 (PMC4227649; doi:10.1371/journal.pbio.1001995)

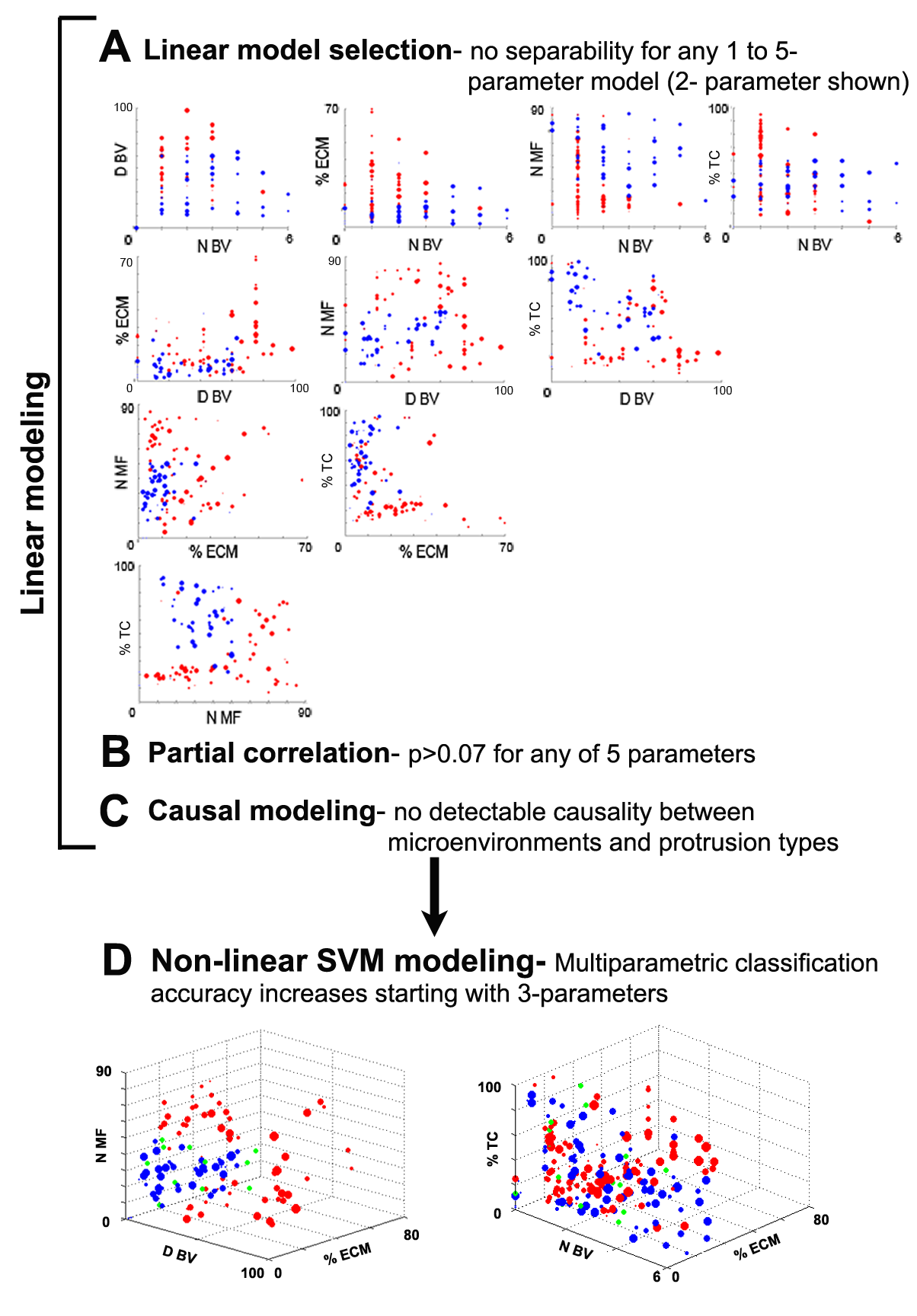

Supplement: Figure S1 — Distinguishing between tumor microenvironment conditions where fast- or slow-locomotion occurs can not be done with linear models but with non-linear SVM modeling. (A) Model selection using any of 1, 2, 3, 4, or 5 microenvironment parameters does not provide separability of microenvironments in which fast locomotion (blue) or slow-locomotion (red) occur. As an illustration, all 2-parameter scatter plots are shown. There are no lines that can be drawn to separate blue and red points without significant misclassification. N MF, Number of macrophages; N BV, Number of blood vessels; D BV, diameter of blood vessels; % ECM, area covered by collagen fibers; % TC, area covered by tumor cells. (B, C) Partial correlation or causal modeling do not yield statistically significant results. (D) Non-linear classification using SVM methodology resulted in 92% accuracy, with only a few misclassified points. Accuracy is based on 10-fold cross-validation. Presented here are two SVM classifications based on three out of the five microenvironmental parameters. On the left is a classification based on D BV, N MF, and % ECM, yielding 84% accuracy; on the right, classification is based on N BV,% ECM, and % TC, yielding 78% accuracy. Misclassified data (green points) are tumor cells whose observed class of protrusion is opposite to that predicted by the SVM classification algorithm. Numerical values used for analysis are the same as in Figure 2C. (TIF) [file pbio.1001995.s001.tif]

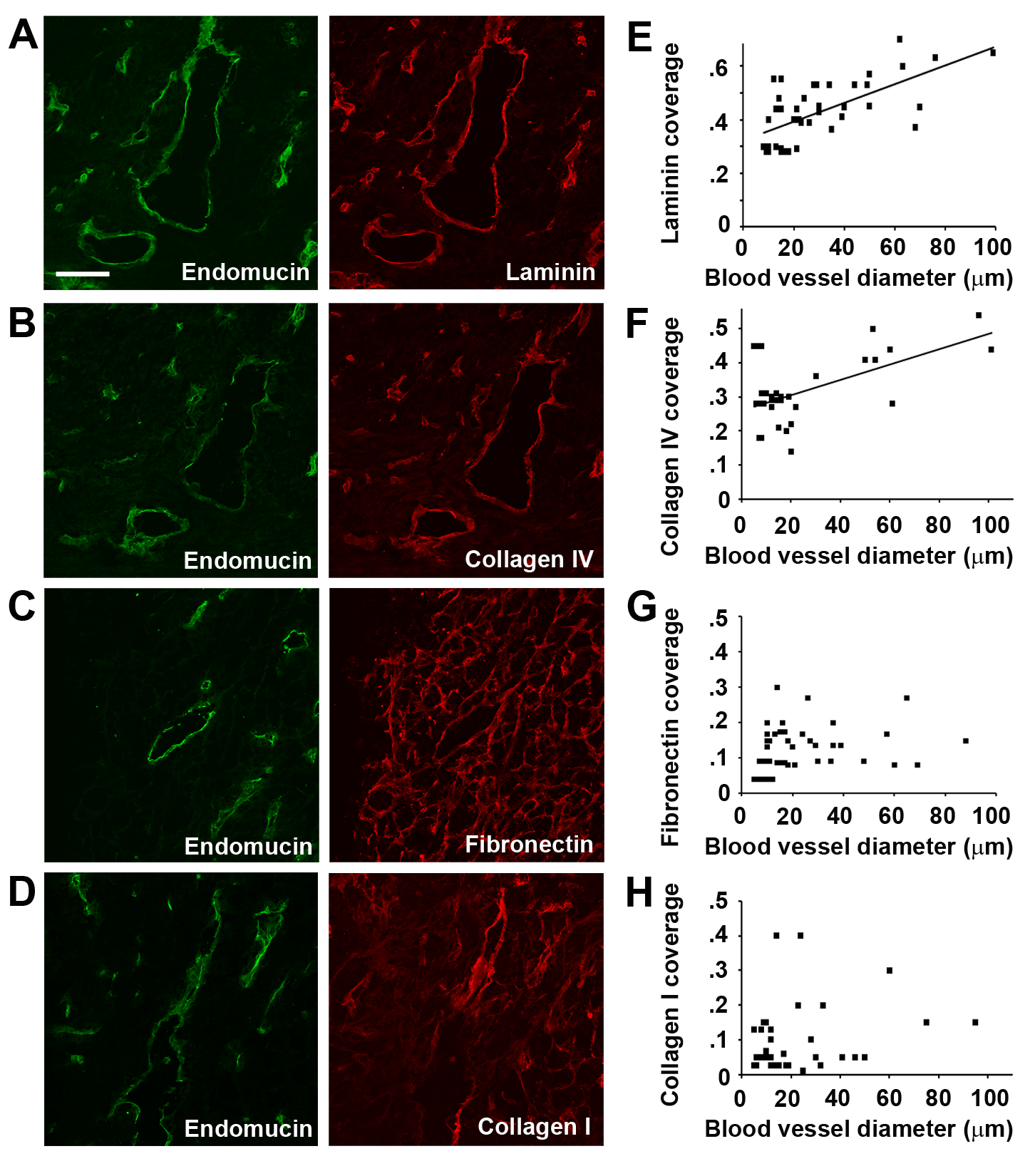

Supplement: Figure S2 — Correlation of blood vessel diameter and blood vessel coverage with ECM components. (A–D) Blood vessels were visualized by endomucin (green) and coverage of the blood vessel was measured using laminin (red) (A), collagen I (B), fibronectin (C), and collagen I (D). Scale bar: 50 µm. (E–H) We subsequently plotted blood vessel diameter versus coverage by ECM components. While the coverage of basement membrane proteins increases linearly with blood vessel diameter (E, F), fibronectin and collagen I levels are not linked to the size of blood vessels and are present in both the perivascular and interstitial ECM (G, H). (TIF) [file pbio.1001995.s002.tif]

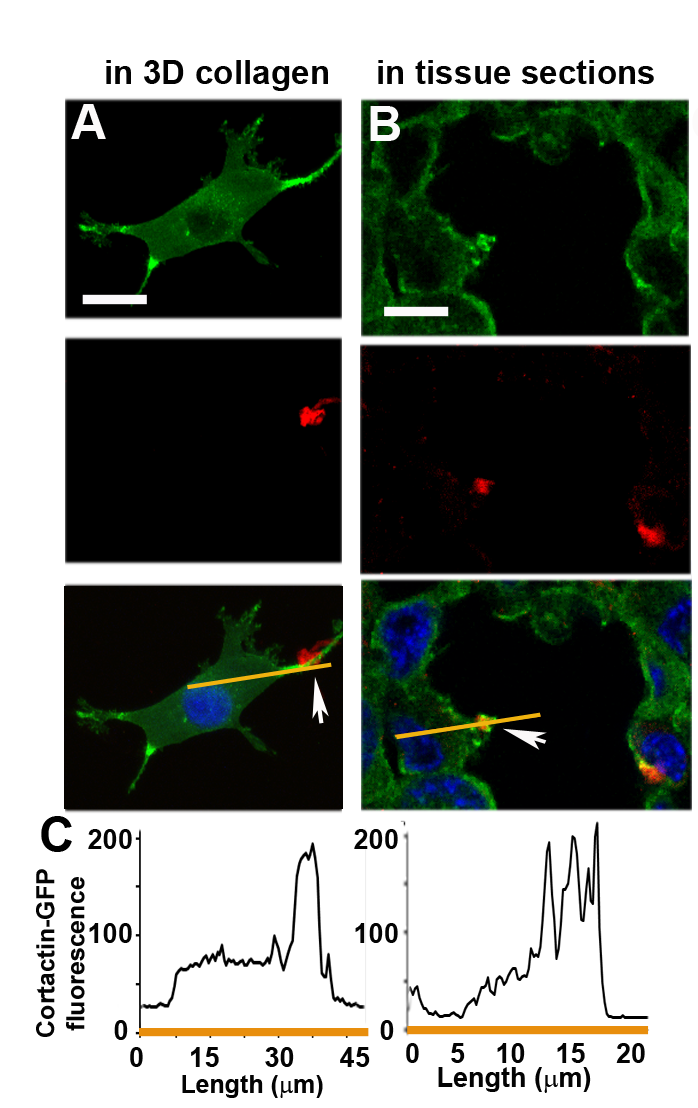

Supplement: Figure S3 — Cortactin is enriched at the tip of collagen-degrading protrusions in 3-D culture and tissue sections. (A) In 3-D culture of MDA-MB-231-cortactin-GFP cells, cortactin (green) is enriched at the tip of the small protrusion, which is surrounded by degraded collagen (red, Col 2 ¾ C antibody). Cell nucleus is labeled by DAPI (blue). Scale bar: 5 µm. (B) In tissue cryosections of MDA-MB-231-cortactin-GFP tumors, cortactin is similarly enriched at the tips of small protrusions in perivascular tumor cells and degraded collagen surrounds cortactin enrichment. Scale bar: 10 µm. Arrows point to tips of protrusions. (C) Line-scans measuring cortactin-GFP along yellow lines in (A and B). Lines go through protrusions and show peaks of cortactin concentration at the tips. (TIF) [file pbio.1001995.s003.tif]

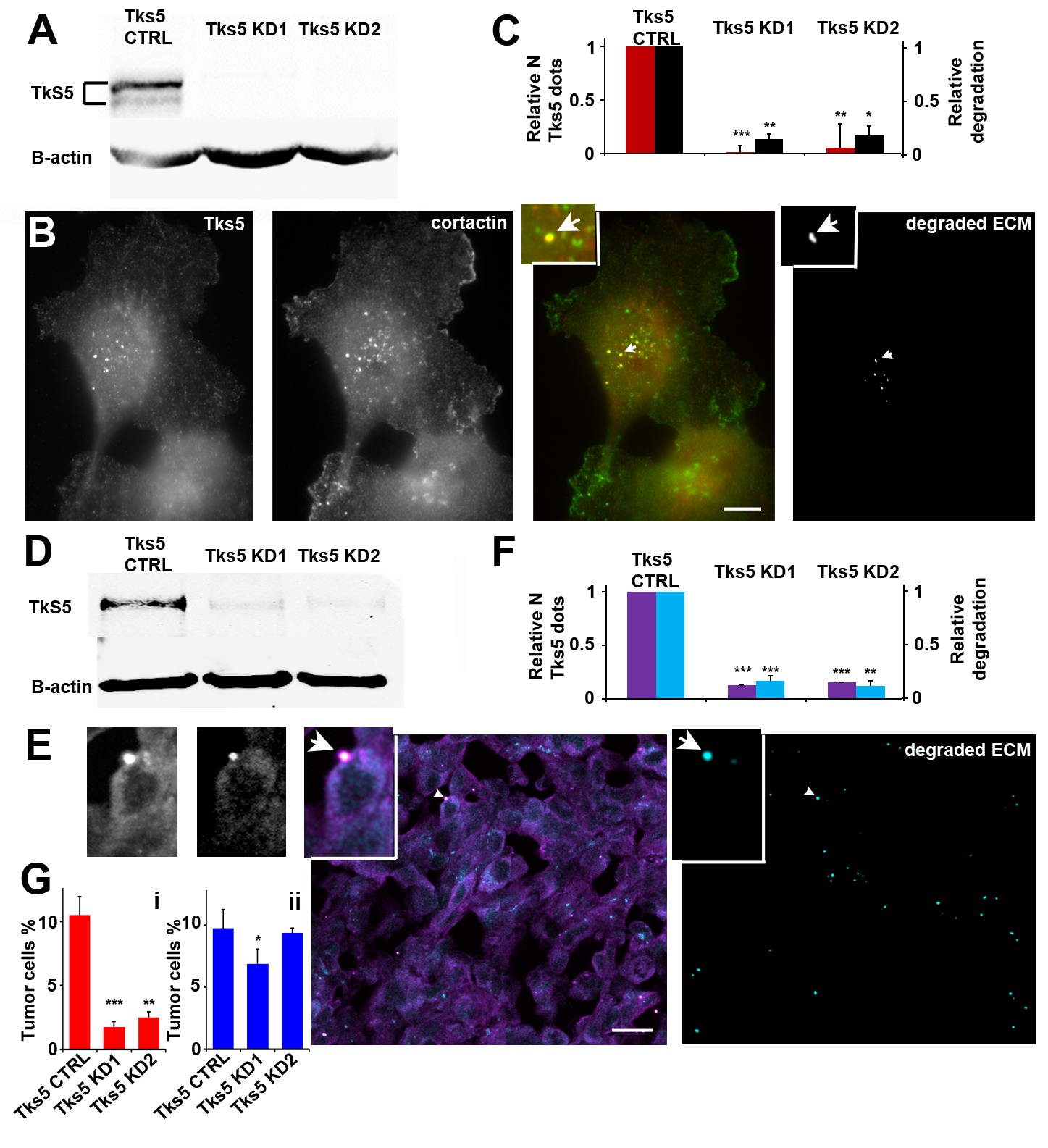

Supplement: Figure S4 — Tks5 knockdown inhibits appearance of invadopodia both in vitro and in the primary tumor. (A) Western blot showing Tks5 levels in control plKO.1 cell line transduced with non-targeted shRNA and Tks5 knockdown cell lines. Knockdown efficiency is 96% and 99% for Tks5 KD1 and Tks5 KD2, respectively. (B) Representative images of Tks5 CTRL cells plated on fluorescent gelatin and labeled with Tks5 (first panel) and cortactin (second panel) antibodies. Color panel shows overlay of Tks5 (red) and cortactin (green); insert zooms in to an invadopodium in vitro (arrow). Right panel shows corresponding holes in fluorescent gelatin (white), created by invadopodia (arrow). Bar 5 µm. (C) Quantification of images shown in (B). Numbers of Tks5 dots (red bars) and total area of gelatin degraded over 6 h (black bars) in the control and knockdown cell lines. Bars represent means ± SEM of n = 4 dishes, ran as duplicates, each with ten to 15 fields of view. (D) Western blot showing levels of Tks5 in tumor cells sorted from the primary tumor. Knockdown efficiency is 89% and 95% for Tks5 KD1 and Tks5 KD2, respectively. (E) Representative cryosection of Tks5 CTRL primary tumor with Tks5 (left insert) and degraded collagen I (middle insert) antibodies. Color panel shows overlay of Tks5 (purple) and degraded collagen I (cyan); color insert zooms in to an invadopodium ex vivo (arrow). Right panel shows degraded collagen I (cyan), created by invadopodia (arrow). Bar 20 µm. (F) Quantification of images shown in (E). Numbers of Tks5 dots (purple bars) and total area of degraded collagen I (cyan bars) in the control and knockdown cell lines. Bars represent means ± SEM of ten to 15 fields of view. (G) The fraction of tumor cells within the same field of view which exhibit small protrusions in vivo and associated slow-locomotion (red, panel i) is dramatically reduced in Tks5 knockdown tumors compared to Tks5 CTRL tumors. In the same tumors, fractions of tumor cells exhibiting fast-locomotion (blue, [file pbio.1001995.s004.tif]

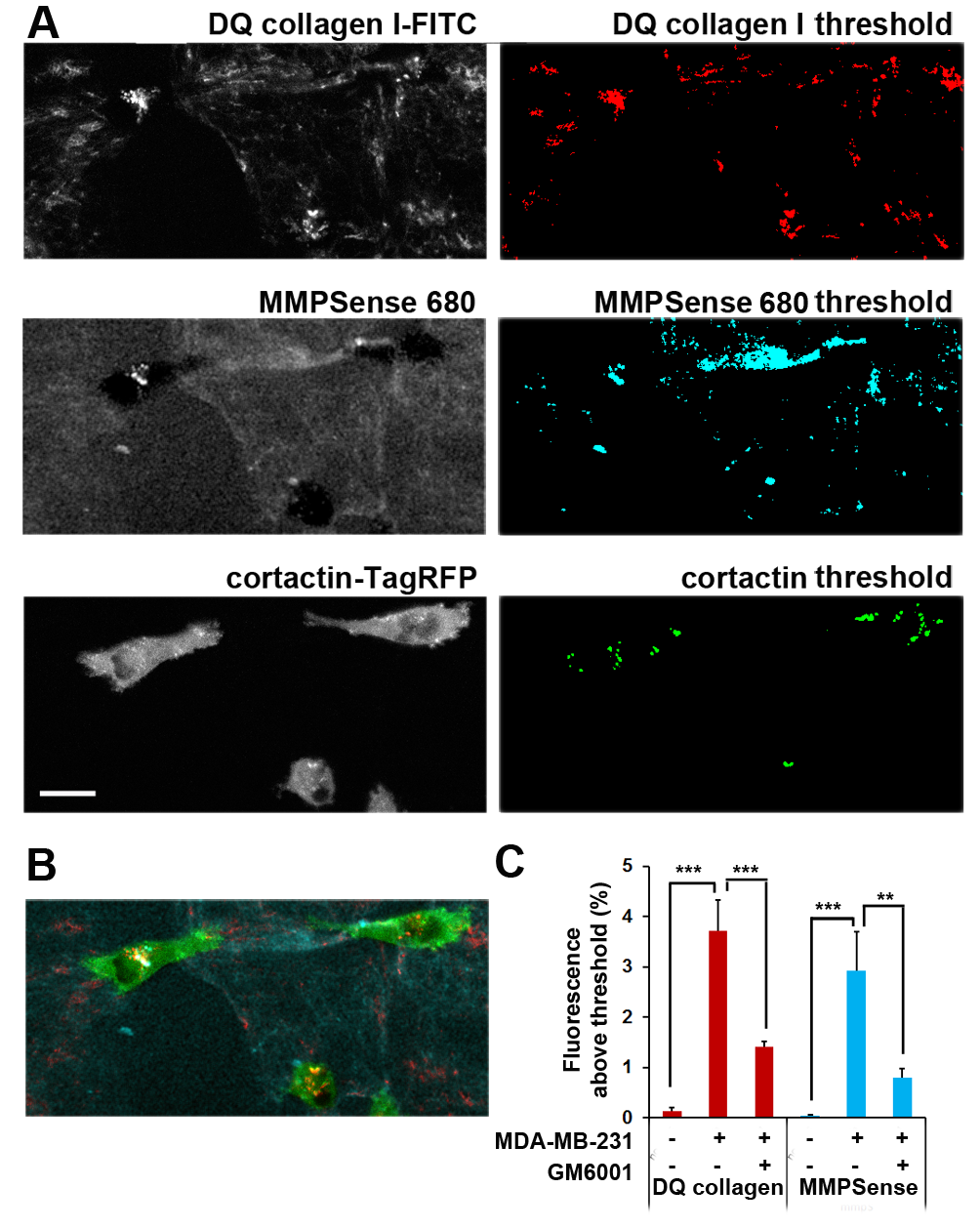

Supplement: Figure S5 — Comparison of localization and signal trends of the MMPSense 680 fluorescent reporter of ECM remodeling and degradation with established reporter DQ collagen I in 3-D culture. (A) Raw (left) and thresholded images (right) of DQ collagen I (red) and MMPSense 680 (cyan) fluorescent signals at 6 h after addition into MDA-MB-231-cortactin-TagRFP (green) in 3-D collagen. DQ collagen I gel was mixed with collagen I 1∶10 and MMP Sense 680 solution was mixed with culture media 1∶100. Optimal threshold levels were determined by averaging images taken at 0 h (Materials and Methods). Maximum projection of 5 µm total is shown. Scale bar: 15 µm. (B) Overlay image of DQ-collagen I, MMPSense 680, and tumor cells shows that DQ-collagen I and MMPSense 680 signals have similar localization, surrounding motile cells, with MMPSense 680 signal being more diffuse. (C) Fluorescence above threshold in the control gel or in the presence of tumor cells with or without treatment with 25 µM GM6001. Fluorescent signal indicating degraded ECM was significantly increased in the presence of tumor cells, but was reduced when those cells were treated with GM6001. The same trends were observed with both DQ collagen I and MMPSense 680. **p<0.01, ***p<0.001, using homoscedastic t-test. Bars represent means of 10–15 fields of view ± SEM; n dishes = 3, done on separate days. (TIF) [file pbio.1001995.s005.tif]

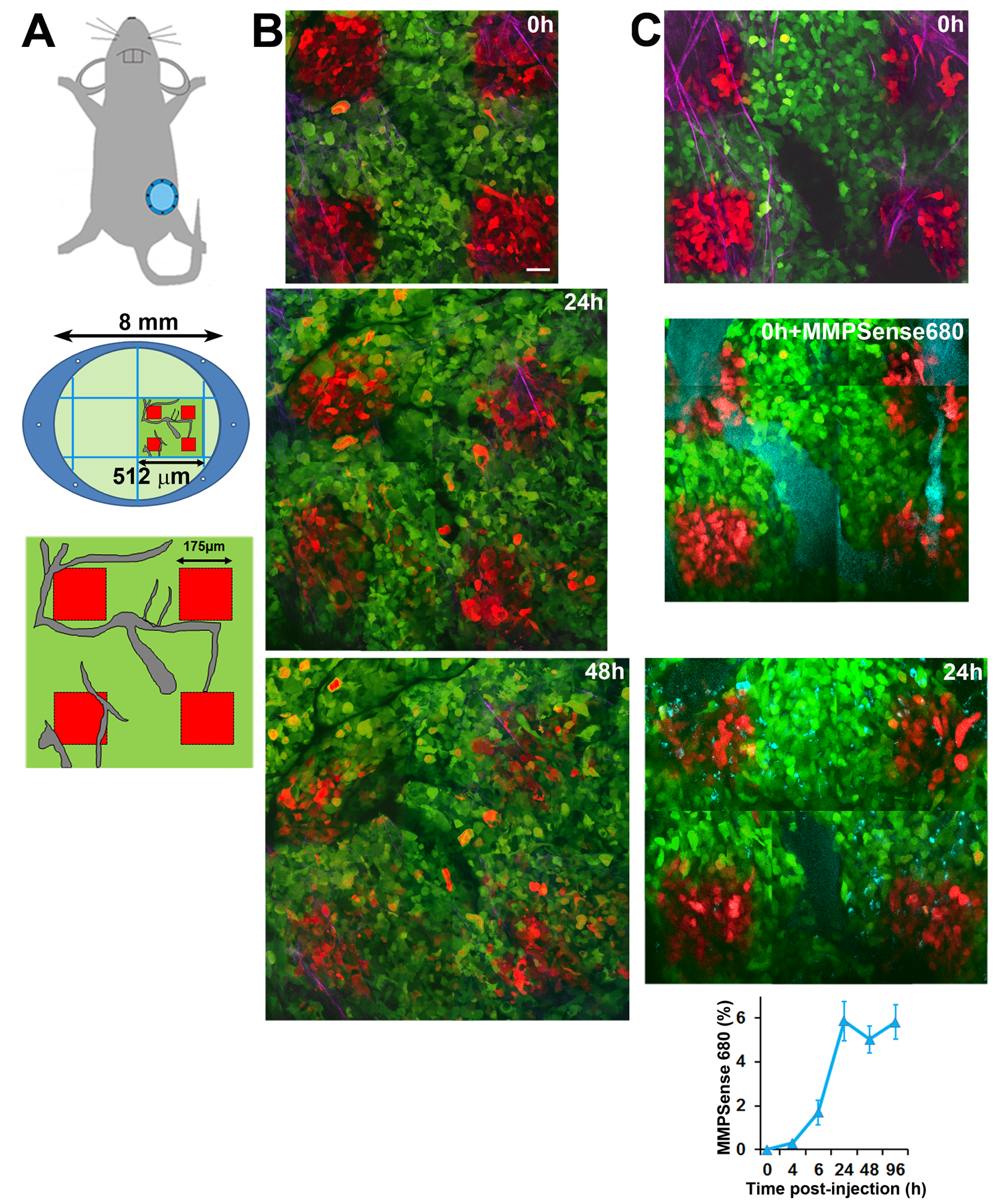

Supplement: Figure S6 — Photoconversion-enabled tumor cell fate-mapping experiment designs. (A) Photoconversion illustration: mammary imaging window implanted on top of the primary tumor contains a round glass coverslip 8 mm in diameter. Four to 12 512×512×100 µm stacks are recorded within the same animal, each one containing a 175×175 µm photoconverted region. (B) Tumor cell fate-mapping in four tiled regions: photoconverted cells (red), non-photoconverted cells (green), and collagen (purple) are monitored at 0, 24, and 48 h. To detect all red cells that dispersed from the original region over time, visualized areas at 24 h and 48 h are increased by recording four separate stacks and stitching them into a mosaic stack. Images shown are maximum projections of ten sections combined to 50 µm in depth. Scale bar: 50 µm. (C) Photoconversion followed by MMPsense 680 injection: tumor cells are photoconverted at 0 h (top panel), followed by MMPSense 680 tail-vein injection (MMPSense 680, cyan; collagen was excluded for clarity), which extravasates and accumulates in perivascular regions, locating spots of ECM degradation. Maximum projections of total 50 µm in depth are shown. Graph shows how normalized MMPSense 680 signal increases over time and plateaus at 24 h (see Materials and Methods). (TIF) [file pbio.1001995.s006.tif]

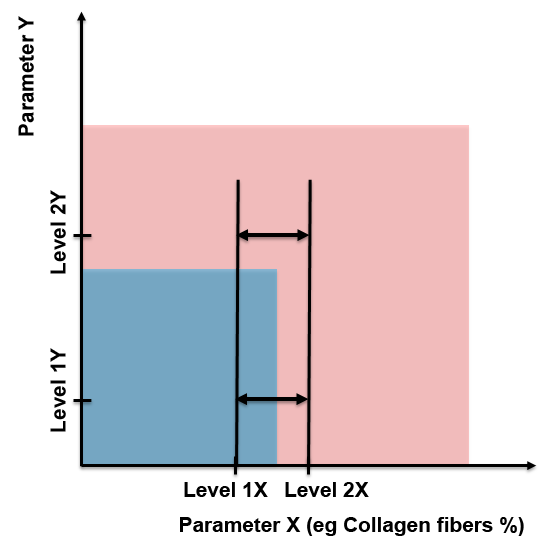

Supplement: Figure S7 — Illustration of context dependency principle. A shift from level 2X to level 1X in the parameter X will cause a phenotypic switch from slow-locomotion (red) to fast locomotion (blue) motility when parameter Y is at level 1Y, but not when parameter Y is at level 2Y. In the experiment shown in Figure 5, parameter X is collagen fiber density, which we manipulated by changing ECM cross-linking using ribose or BAPN. Parameter Y may be any of the other four parameters (number of macrophages or blood vessels, blood vessel diameter, or tumor cell crowding). (TIF) [file pbio.1001995.s007.tif]

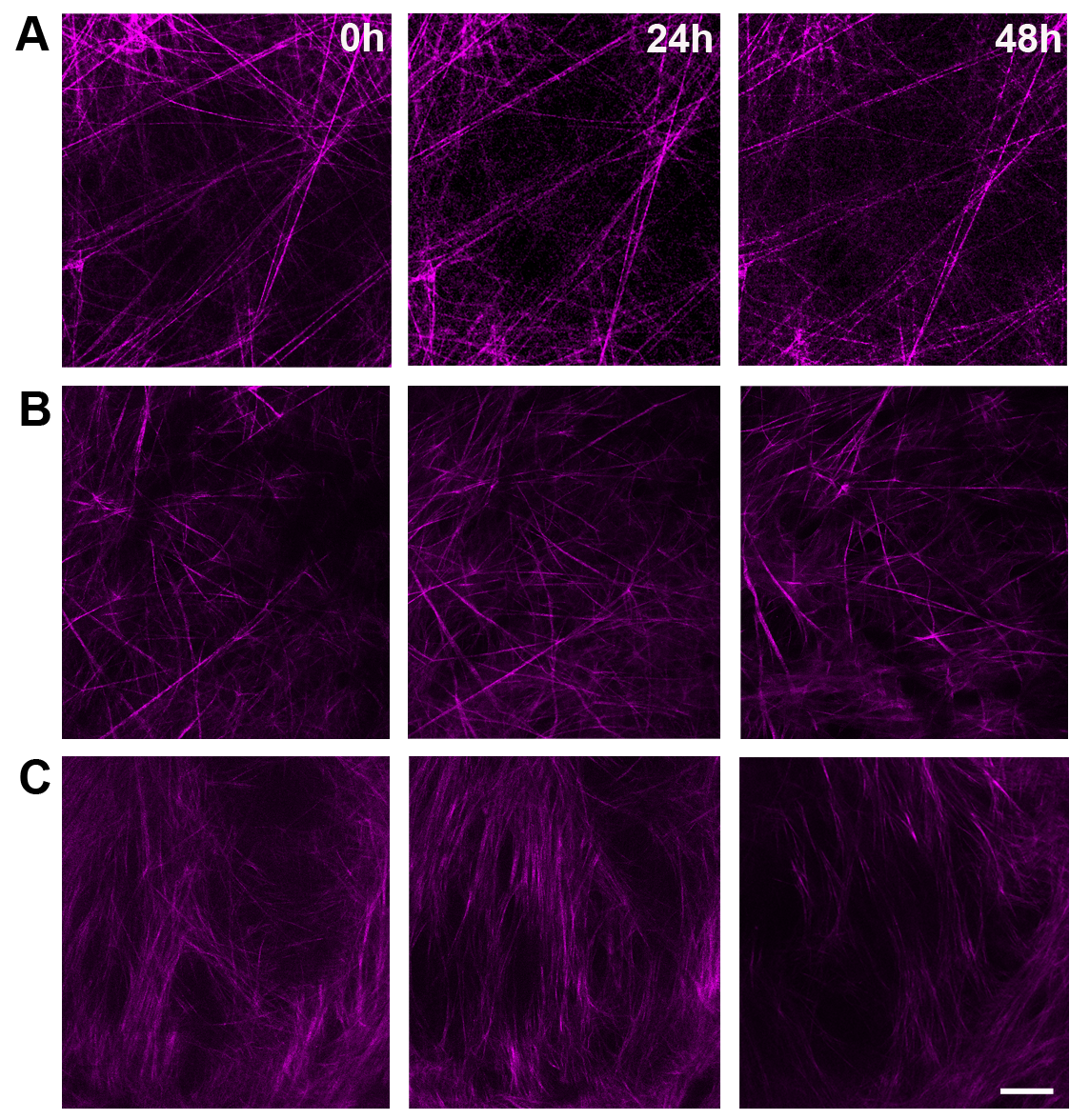

Supplement: Figure S8 — Collagen fiber density in vivo modulation by treatment with L-ribose or BAPN. (A) Collagen fibers imaged in the same field of view at 0, 24, and 48 h. (B) Collagen fibers imaged 0–48 h in an animal treated with 1 g/kg/day L-ribose. (C) Collagen fibers imaged 0–48 h in an animal treated with 6 mg/kg/day lysil oxidase (LOX) inhibitor BAPN. Scale bar: 75 µm. (TIF) [file pbio.1001995.s008.tif]

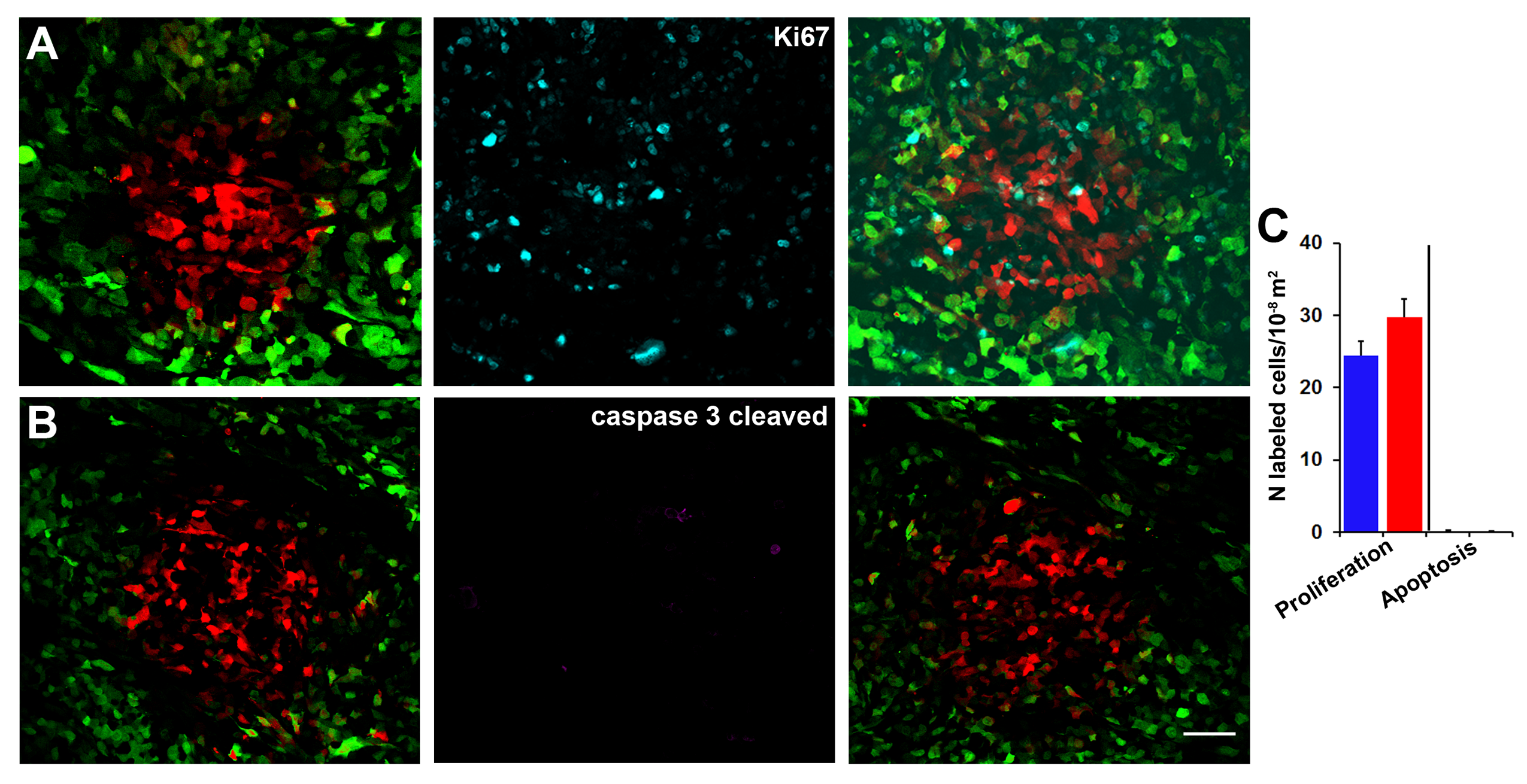

Supplement: Figure S9 — Proliferation and apoptosis rates are similar in fast- and slow-locomoting photoconverted cells. (A) Proliferation in areas of fast- and slow-locomotion: Representative image of tumor cells in photoconverted area visualized via multiphoton microscopy in vivo at 24 h (green, non-photoconverted cells; red, photoconverted cells, left panel); ex vivo, same area visualized via confocal microscopy after cryopreservation and staining with Ki67 (cyan, middle panel) and ex vivo, same area when Ki67 and tumor cells are overlayed (right panel). (B) Apoptosis in areas of fast- and slow-locomotion: Representative image of tumor cells in photoconverted area visualized via multiphoton microscopy in vivo at 24 h (green, non-photoconverted cells; red, photoconverted cells, left panel); ex vivo, same area visualized via confocal microscopy after cryopreservation and staining with caspase 3-cleaved (purple, middle panel) and ex vivo, same area when caspase 3-cleaved and tumor cells are overlayed (right panel). Scale bar 40 µm. (C) Numbers of proliferating and apoptotic cells (practically none) in the photoconverted areas where fast- (blue bars) or slow-locomotion (red bars) were detected at 0 h. (TIF) [file pbio.1001995.s009.tif]

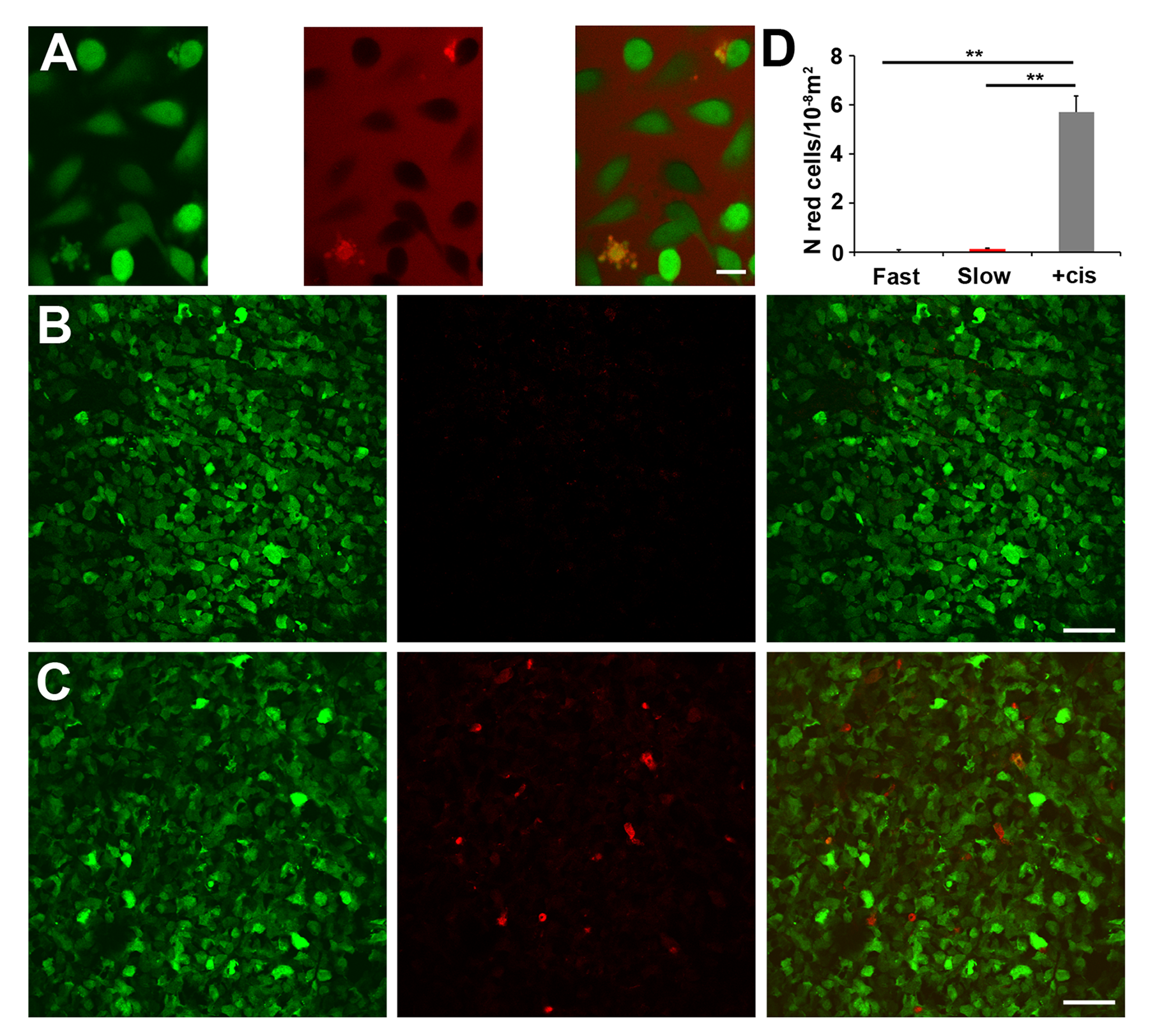

Supplement: Figure S10 — Apoptosis in areas of fast- and slow-locomotion measured directly using SR-FLIVO marker. (A) SR-FLIVO marker mixed in culture media conditions does not label any cells, but in the positive control treated by 50 µM cisplatin for 2 h, it concentrates in apoptotic cells. Tumor cells (green, left), SR-FLIVO in media (red, middle), overlay (right). Bar 20 µm. (B, C) SR-FLIVO marker in vivo does not label any cells in fast- or slow-locomotion areas (B), but concentrates in apoptotic cells induced by 5 day-treatment with 2 mg/kg cisplatin (C). Tumor cells (green, left), SR-FLIVO in vivo (red, middle), overlay (right). Bars 80 µm. (D) Numbers of apoptotic cells in the areas of fast- (blue bars) or slow-locomotion (red bars) were detected or in the animals treated with cisplatin (grey bars).**p<0.01. (TIF) [file pbio.1001995.s010.tif]

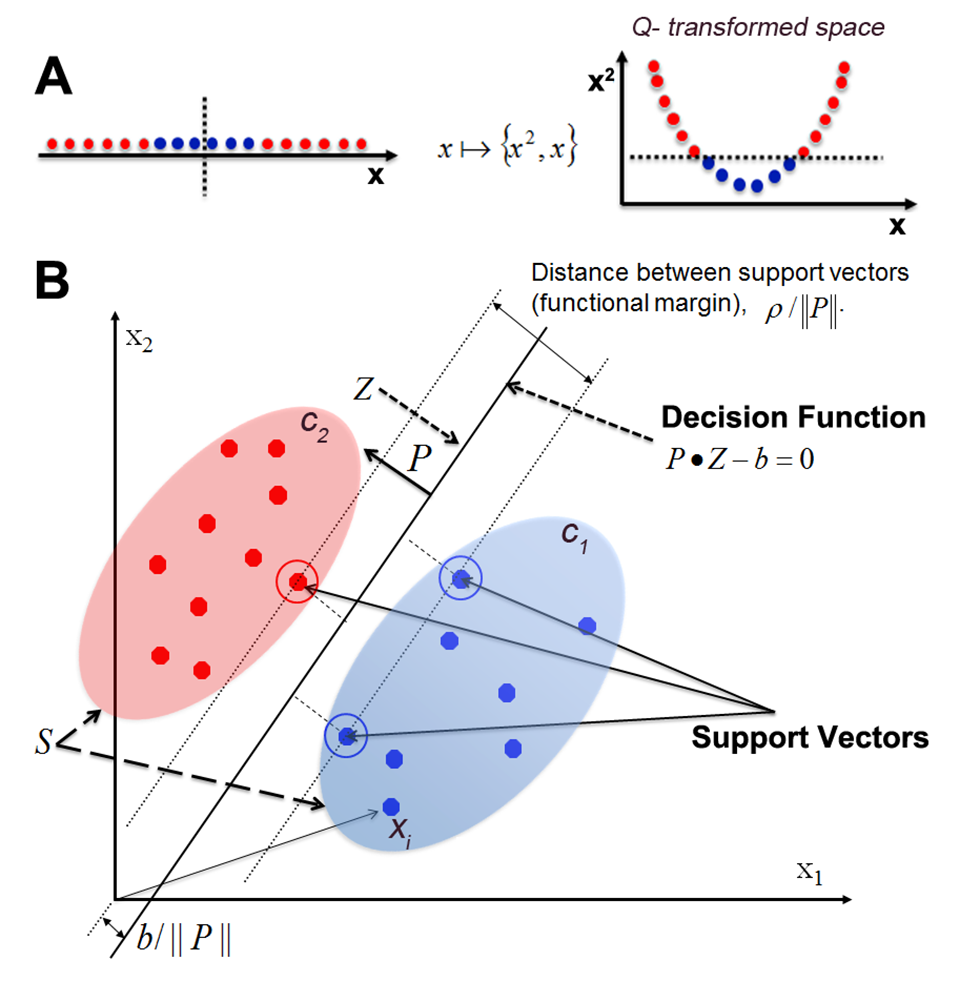

Supplement: Figure S11 — Principle of support vector machine classification. (A) Non-linear transformation of input space increases dimensionality and allows for linear separation. (B) Classification using support vectors to find optimum hyperplane (Z). (TIF) [file pbio.1001995.s011.tif]
